# An MCDM approach based on some new Pythagorean cubic fuzzy Frank Muirhead mean operators

**DOI:** 10.1016/j.heliyon.2022.e12249

**Published:** 2022-12-06

**Authors:** Pankaj Kakati

**Affiliations:** Department of Mathematics, Jagannath Barooah College, Jorhat-785001, India

**Keywords:** Pythagorean cubic fuzzy set, Frank operations, Muirhead means, MCDM

## Abstract

Pythagorean cubic fuzzy sets (PCFSs) are the most convenient aid to depict ambiguous data in practical decision-making situations. The Muirhead mean (MM) possesses the ability to capture the interrelationship between the criteria. Further, the MM generalizes several significant operators, for instance, the Bonferroni mean (BM) and the Maclaurin symmetric mean (MSM), etc. The Frank triangular norms can offer significant adaptability and robustness due to the presence of an additional parameter as compared to other families of triangular norms. Therefore, Muirhead mean and Frank operations can be combined for obtaining suitable results during decision-making under Pythagorean cubic fuzzy information with interrelated criteria. The primary goal of this study is to introduce some new MM operators based on Frank operations, namely, PCFFMM and PCFFGMM. Then some of the properties of PCFFMM and PCFFGMM are described. Moreover, an MCDM approach is formulated using PCFFMM and PCFFGMM operators. Eventually, the projected MCDM approach is demonstrated with a numerical example. The phenomenon of interrelationships among criteria of real-life problems can be suitably handled by the PCFFMM and PCFFGMM. The overall ranking result obtained by utilizing the proposed operators PCFFMM or PCFFGMM is more appropriate as compared to the ones obtained by using the BM, MSM, Heronian mean (HM), Choquet integral (CI), etc. Moreover, PCFFMM or PCFFGMM can be preferred ahead of BM, MSM, HM, and CI due to its computation simplicity, accuracy, flexibility, and robustness during the aggregation process of multiple correlated input data.

## 1 Background study

The fuzzy set (FS) [1] is an effective aid for expressing the imprecise and vague information involved within real-life decision-making problems. An FS is represented by the grade membership, which represents the index of approval of information. However, the fuzzy set [1] is unable to interpret the situation of non-acceptance or rejection of information. To handle this, Atanassov [2], [3] proposed the intuitionistic fuzzy sets (IFS), by allocating a non-membership grade in addition to the grade of membership. The IFS has been widely utilized to model several MCDM models [4], [5], [6], [7], [8], [9], [10], [11], [12], [13]. Tao et al. [13] provided an algorithm for dynamic group MCDM adapted from the alternative queuing model using intuitionistic fuzzy data by introducing an induced ordered weighted average operator. Verma [14] proposed a generalized weighted BM and developed a MADM method for IFS data. Gu et al. [15] proposed a TOPSIS approach to evaluate the risk index of landslide hazards in Shiwangmiao city under IFS information. However, in many real-life problems, the aggregate of grades of membership and non-membership of element possibly bigger than 1, instead their square sum is either less than or equal to 1. Yager [36], [37], [38], [39] introduced the Pythagorean fuzzy set (PFS) which is described by the grades of membership and non-membership such that their square sum is either less than or equal to 1. Further, Yager [36], [37] proposed several Pythagorean fuzzy weighted operators. Peng and Yeng [40] introduced several novel operations on PFSs. Zang and Xu [41] provided an generalization of the TOPSIS on PFSs. Zeng et al. [42] defined several weighted average operators based on average distance and developed a hybrid TOPSIS method on PFSs. Ren et al. [43] proposed the TODIM method for PFSs. Garg [44] proposed some Pythagorean fuzzy Einstein weighted aggregation operators for MCDM. Naz et al. [45] defined the Pythagorean fuzzy graph (PFG) and the Pythagorean fuzzy preference relations (PFPRs) and developed a novel MCDM approach. Li and Zeng [46] presented some distance measures for PFSs and PFNs. Xiao and Ding [47] introduced a new Jensen–Shannon distance measure under PFS and developed a method for medical diagnosis. Verma and Merigó [48] introduced the extended cosine and cotangent similarity measures for PFSs. Bakioglu and Atahan [49] introduced an MCDM model adapted from the AHP, TOPSIS, and VIKOR methods under the PFS environment for the risk assessment of self-driving vehicles. Recently, several MCDM approaches [49], [50], [51], [52], [53], [54], [55], [56], [57], [58], [59], [60], [61] have been designed on PFSs. Gündoğdu and Kahraman [17] defined the spherical fuzzy set (SFS) which is expressed by the three-dimensional membership grades to express ambiguous information involving several different opinions of the type yes, abstain, no and refusal, etc. Perveen et al. [18] combined the SFS and soft set to propose the spherical fuzzy soft set (${\mathrm{SFS}}_{ft}S$). Ahmmad et al. [19] formulated several aggregation operators and developed an MCDM method under the ${\mathrm{SFS}}_{ft}S$. Iampan et al. [21] proposed some linear Diophantine fuzzy Einstein operators and introduced an MCDM method under the linear Diophantine fuzzy set (LDFS) [20] which can overcome the restrictions present in IFS, PFS, and q-ROFS. Águila et al. [22] introduced a multicriteria decision-making approach for studying responsive power inside Electric Microgrids.

Nevertheless, in some practical situations because of lack of data and time, it becomes challenging for DMs to represent the membership grades of an element into an exact numeric value. To overcome this, Zhang [62] extended PFS to the interval-valued Pythagorean set (IVPFS) that allows the grades of membership and non-membership of an element to interval values in [0,1]. Peng and Yang [64] proposed several weighted aggregation operators under the IVPFSs. Garg [65] introduced some exponential operational rules on IVPFS and developed several weighted exponential aggregation operators on IVPFS. Further, Garg [66] introduced an enhanced accuracy function for comparing IVPFSs. Khan and Abdullah [67] introduced an Choquet integral average and extended the conventional GRA method to a novel GRA method under the IVPFS information. For further investigation on IVPFSs, we may refer to [68], [69], [70], [71], [72], [73]. Both PFS and the IVPFS are very effective to address uncertainty and imprecision involved in practical problems. Most of the existing decision-making approaches have been investigated by ignoring the confidence levels of the criteria and assuming that DMs are acquainted with the evaluated result of MCDM problems. However, in practical decision-making problems, confidence levels can affect the overall outcome. To overcome this, Garg [63] integrated the concept of the confidence levels with PFSs during the evaluation of MCDM problems.

### 1.1 Related works

Khan et al. [74] extended the IVPFS to the Pythagorean cubic fuzzy (PCFS) for which the square sum of the supremum of its grades of membership is either smaller or equal to 1. Khan et al. [74], further proposed several weighted operators and developed an MCDM method under PCFS. Khan et al. [75] defined some averaging and geometric operators on PCFS by taking into account the confidence levels of DM's during evaluation stages. Hussain et al. [78] introduced some new operational laws on PCFS and developed some Pythagorean cubic fuzzy Einstein weighted operators. Wang and Zhao [76] formulated some distance measurements and developed an MCDM approach on the PCFSs. Talukdar and Dutta [77] introduced a class of distance measures on cubic PFSs and studied medical MCDM problems.

Most of the aforementioned operators are based upon either Algebraic or Einstein triangular norms which provide less flexibility and robustness during the process of aggregation. The Frank triangular norms [79] proposed by Frank is a flexible class of continuous *t*-norms that involve an additional parameter, that provides more flexibility during the process of aggregation. Calvo et al. [80] investigated the Frank and Alsina functional equations for two families of commutative, associative, and increasing binary operators. Sarkoci [81] provided an investigation on the domination of the classes of Frank and Hamacher *t*-norms. Recently, several Frank aggregation operators [82], [83], [84], [85], [86], [87], [88], [89], [90], [91] have been proposed using the Frank triangular norms for MCDM problems.

In most of the practical-world problems, there often exists dependency among the criteria of the problem. Therefore, the interaction among the criteria should be considered while obtaining the final evaluation result. Several operators like the Bonferroni mean (BM) [23], Heronian mean (HM) [24], Choquet integral (CI) [92] and Muirhead mean (MM) [25] can reflect the inter-relationship between the input arguments. Nevertheless, Liu and Li [26] described some advantages of Muirhead mean over BM and HM. Although, the CI [92] with respect to fuzzy measure [93] is a useful tool to represent inter-relationship between the criteria, yet it has the computational difficulty of solving fuzzy measures, Muirhead mean doesn't involve such types of difficulties. Qin and Liu [27] introduced the 2-tuple linguistic MM and developed a MAGDM model for supplier selection. Li et al. [28] proposed some new Pythagorean fuzzy power MM operators and introduced a novel MADM method. Liu et al. [29] introduced some MM to solve MADM problems under the PFLS environment. Yang and Chang [30] introduced the interval q-ROF weighted power MM operator and developed an evaluation model for garbage disposal site selection. Garg [31] introduced several cubic q-ROF linguistic MM operators and developed a MADM method under the Cq-ROFLS environment.

### 1.2 Motivation

In view of the above review, the following can be summarized as the main reasons for the current investigation.

1. The PCFS [74] being a generalization of the IVPFS [62] is an efficacious tool to express the uncertainty and impreciseness of real-life decision-making problems more appropriately than FS, IFS, etc. Pythagorean cubic fuzzy sets can take into consideration the confidence levels [75] of each DM during the assessment process. Recently, several MCDM methods [75], [76], [77], [78] have been proposed on the PCFSs. Operators defined on PCFS are more efficient and flexible as compared to the operators based on other extensions of fuzzy sets. Moreover, the PCFS can provide more accurate results due to the ability to handle uncertainty, perspicacity, and prejudice of decision-makers (DMs) during complex decision-making problems.
2. Frank triangular norms [79] is a class of continuous triangular norms and are the generalization of the probabilistic and Lukasiewicz triangular norms [16]. Frank operations [79] based on Frank triangular norms are more flexible and robust as compared to the Algebraic operations and the Einstein operations due to the presence of an additional parameter. Applicability of Frank *t*-norms can be seen in various practical decision-making problems [82], [83], [84], [85], [86], [87], [88], [89], [90], [91].
3. Muirhead mean [25] expresses the inter-relationship among several input values and also provides more flexibility and robustness during the aggregation process due to a vector parameter. The BM [23] and MSM [35] are effective tools for dealing with interrelated criteria. However, BM and MSM are some of the particular cases of the MM, hence MM can provide more general and robust results during complex decision-making situations. Moreover, Muirhead mean [25] is preferable to BM and HM as it can reflect the interaction among multiple input values, whereas BM and HM can deal with the interaction among two input values. Furthermore, Muirhead mean [25] can be preferred ahead of the Choquet integral [92] because the MM operator doesn't have the computation complexity of solving fuzzy measures [93] during the assessment of MCDM problems with a large set of criteria. Recently, several MM operators have been introduced in various extensions of fuzzy sets and utilized to solve MCDM problems [26], [27], [28], [29], [30], [31], [32], [33], [34].

Thus motivated by these, it becomes necessary to combine the Muirhead mean [25] with the Frank operations [79] under the PCFS environment to develop the PCFFMM operator. Since there is hardly any study found in the literature on Frank MM operators under the PCFS environment. Hence the present study becomes more appropriate and significant to deal with the interactions among the criteria.

Some of the fundamental objectives of the current study are:

(1) To introduce several operational rules on PCFNs on the base of Frank triangular norms.
(2) To develop some novel Frank Muirhead mean operators such as PCFFMM and PCFFGMM for Pythagorean cubic fuzzy information aggregation with interrelated criteria.
(3) To introduce an MCDM approach utilizing the PCFFMM and PCFFGMM for obtaining the overall evaluation of real-life MCDM problems with Pythagorean cubic fuzzy information.
(4) To present a demonstration and a comparison analysis of the projected MCDM approach.

The study continues in the following manner. Section 2, concisely discusses some fundamental notions, Section 3, introduces some Frank operational rules on the PCFNs. Section 4, proposes the PCFFMM and PCFFGMM operators. Furthermore, some of their special cases and properties are discussed. Section 5, introduces a multicriteria decision-making approach using the PCFFMM and PCFFGMM operators. Section 6, demonstrates the applicability of the projected approach with a numerical example. A parallel study of the projected approach with some existing approach is presented. Section 6, contains the closing statements.

## 2 Fundamentals

This section presents several basic notions of the relevant literature compiled from [2], [3], [36], [37], [38], [74] which will be used for the development of our model.

Definition 2.1 [2], [3] The IFS F on a set V is presented as

$$F=\{\langle v,\mu_{F}(v),\nu_{F}(v)\rangle |v\in V\},$$

here $\mu_{F}(v),\nu_{F}(v)\in [0,1]$ denote the grades of membership and the non-membership of each v∈V to F, and $0⩽\mu_{F}(v)+\nu_{F}(v)⩽1$.

Definition 2.2 [36], [37], [38] The PFS P on a set V is given as

$$P=\{\langle v,\mu_{P}(v),\nu_{P}(v)\rangle |v\in V\},$$

here $\mu_{P}(v),\nu_{P}(v)\in [0,1]$, respectively, represent the grades of membership, and non-membership with $0⩽{\left(\mu_{P}(v)\right)}^{2}+{\left(\nu_{P}(v)\right)}^{2}⩽1$ for each v∈V. Also, the grade of indeterminacy is $\pi_{P}(v)=\sqrt{1-{\left(\mu_{P}(v)\right)}^{2}-{\left(\nu_{P}(v)\right)}^{2}}$. The pair $p=\langle \mu_{p},\nu_{p}\rangle$ is identified as a PFN [41].

Several fundamental operational rules on PFNs are as follows [41]:

Definition 2.3 [41] Suppose $p=\langle \mu_{p},\nu_{p}\rangle$ and $q=\langle \mu_{q},\nu_{q}\rangle$ be two PFNs, and then the following operations hold:

(i) $p⊕q=\langle \sqrt{\mu_{p}^{2}+\mu_{q}^{2}-\mu_{p}^{2}\mu_{q}^{2}},\nu_{p}\nu_{q}\rangle$,
(ii) $p⊗q=\langle \mu_{p}\mu_{q},\sqrt{\nu_{p}^{2}+\nu_{q}^{2}-\nu_{p}^{2}\nu_{q}^{2}}\rangle$,
(iii) $kp=\langle \sqrt{1-{\left(1-\mu_{p}^{2}\right)}^{k}},\nu_{p}^{k}\rangle$,
(iv) $p^{k}=\langle \mu_{p}^{k},\sqrt{1-{\left(1-\nu_{p}^{2}\right)}^{k}}\rangle$

Definition 2.4 [62] The IVPFS on a set V is given as

$$\overset{˜}{P}=\{\langle v,\mu_{\overset{˜}{P}}(v),\nu_{\overset{˜}{P}}(v)\rangle |v\in V\},$$

also, $\mu_{\overset{˜}{P}}(v)=[\mu_{\overset{˜}{P}}^{L}(v),\mu_{\overset{˜}{P}}^{U}(v)]\subset [0,1],\nu_{\overset{˜}{P}}(v)=[\nu_{\overset{˜}{P}}^{L}(v),\nu_{\overset{˜}{P}}^{U}(v)]\subset [0,1]$ are respectively, the interval-valued grades of membership and non-membership with $\mu_{\overset{˜}{P}}^{L}(v)=\inf\mu_{\overset{˜}{P}}(v)$ and $\mu_{\overset{˜}{P}}^{U}(v)=\sup\mu_{\overset{˜}{P}}(v)$, similarly $\nu_{\overset{˜}{P}}^{L}(v)=\inf\nu_{\overset{˜}{P}}(v)$ and $\nu_{\overset{˜}{P}}^{U}(v)=\sup\nu_{\overset{˜}{P}}(v)$ such that $0⩽{\left(\mu_{\overset{˜}{P}}^{U}(v)\right)}^{2}+{\left(\nu_{\overset{˜}{P}}^{U}(v)\right)}^{2}⩽1$ for each v∈V. Also, the grades of indeterminacy are $\pi_{\overset{˜}{P}}(v)=[\pi_{\overset{˜}{P}}^{L}(v),\pi_{\overset{˜}{P}}^{U}(v)]$ where $\pi_{\overset{˜}{P}}^{L}(v)=\sqrt{1-{\left(\mu_{\overset{˜}{P}}^{U}(v)\right)}^{2}-{\left(\nu_{\overset{˜}{P}}^{U}(v)\right)}^{2}}$ and $\pi_{\overset{˜}{P}}^{U}(v)=\sqrt{1-{\left(\mu_{\overset{˜}{P}}^{L}(v)\right)}^{2}-{\left(\nu_{\overset{˜}{P}}^{L}(v)\right)}^{2}}$ for all v∈V. For convenience, the pair $\overset{˜}{P}=\langle [\mu_{\overset{˜}{P}}^{L},\mu_{\overset{˜}{P}}^{U}],[\nu_{\overset{˜}{P}}^{L},\nu_{\overset{˜}{P}}^{U}]\rangle$ is identified as an IVPFN.

Khan et al. [74] proposed the PCFS as an extension of the IVPFS.

Definition 2.5 [74] The Pythagorean cubic fuzzy set (PCFS) *C* on V is presented as

$$C=\{\langle v,\mu_{C}(v),\nu_{C}(v)\rangle |v\in V\},$$

here $\mu_{C}(v)=\langle A_{C}(v),\xi_{C}(v)\rangle ,\;\nu_{C}(v)=\langle {\overset{˜}{A}}_{C}(v),\eta_{C}(v)\rangle$ represents the grades of membership and non-membership of each v∈V to *C* such that

$$0⩽{\left(\sup(A_{C}(v))\right)}^{2}+{\left(\sup({\overset{˜}{A}}_{C}(v))\right)}^{2}⩽1,0⩽{\left(\xi_{C}(v)\right)}^{2}+{\left(\eta_{C}(v)\right)}^{2}⩽1.$$

Also, the grade of indeterminacy of PCFS is defined as

$$\pi_{C}(v)=\langle \sqrt{1-{\left(\sup(A_{C}(v))\right)}^{2}-{\left(\sup({\overset{˜}{A}}_{C}(v))\right)}^{2}},\sqrt{1-{\left(\xi_{C}(v)\right)}^{2}-{\left(\eta_{C}(v)\right)}^{2}}\;\rangle .$$

For convenience, the pair $c=(\mu_{c},\nu_{c})=\left(\langle A_{c},\xi_{c}\rangle ,\langle {\overset{˜}{A}}_{c},\eta_{c}\rangle \right)$ is known as Pythagorean cubic fuzzy number (PCFN).

Khan et al. [74] introduced some operational laws for Pythagorean cubic fuzzy numbers.

Definition 2.6 [74] Let $c_{1}=(\langle A_{c_{1}},\xi_{c_{1}}\rangle ,\langle {\overset{˜}{A}}_{c_{1}},\eta_{c_{1}}\rangle )$ and $c_{2}=(\langle A_{c_{2}},\xi_{c_{2}}\rangle ,\langle {\overset{˜}{A}}_{c_{2}},\eta_{c_{2}}\rangle )$ be two PCFNs, where

$$\begin{array}{cc} A_{c_{1}}=[\Lambda_{c_{1}},\Gamma_{c_{1}}],\; & {\overset{˜}{A}}_{c_{1}}=[{\overset{˜}{\Lambda }}_{c_{1}},{\overset{˜}{\Gamma }}_{c_{1}}],\\ A_{c_{2}}=[\Lambda_{c_{2}},\Gamma_{c_{2}}],\; & {\overset{˜}{A}}_{c_{2}}=[{\overset{˜}{\Lambda }}_{c_{2}},{\overset{˜}{\Gamma }}_{c_{2}}]. \end{array}$$

Then for any scalar k>0, the following hold:

(i) $\begin{array}{c} c_{1}⊕c_{2}=(\langle \left[\sqrt{\Lambda_{c_{1}}^{2}+\Lambda_{c_{2}}^{2}-\Lambda_{c_{1}}^{2}\Lambda_{c_{2}}^{2}},\sqrt{\Gamma_{c_{1}}^{2}+\Gamma_{c_{2}}^{2}-\Gamma_{c_{1}}^{2}\Gamma_{c_{2}}^{2}}\;\right],\sqrt{\xi_{c_{1}}^{2}+\xi_{c_{2}}^{2}-\xi_{c_{1}}^{2}\xi_{c_{2}}^{2}}\;\rangle ,\langle \left[{\overset{˜}{\Lambda }}_{c_{1}}{\overset{˜}{\Lambda }}_{c_{2}},{\overset{˜}{\Gamma }}_{c_{1}}{\overset{˜}{\Gamma }}_{c_{2}}\right],\eta_{c_{1}}\eta_{c_{2}}\rangle ) \end{array}$
(ii) $\begin{array}{c} c_{1}⊗c_{2}=(\langle \left[\Lambda_{c_{1}}\Lambda_{c_{2}},\Gamma_{c_{1}}\Gamma_{c_{2}}\right],\xi_{c_{1}}\xi_{c_{2}}\rangle ,\langle \left[\sqrt{{\overset{˜}{\Lambda }}_{c_{1}}^{2}{\overset{˜}{\Lambda }}_{c_{2}}^{2}-{\overset{˜}{\Lambda }}_{c_{1}}^{2}{\overset{˜}{\Lambda }}_{c_{2}}^{2}},\sqrt{{\overset{˜}{\Gamma }}_{c_{1}}^{2}+{\overset{˜}{\Gamma }}_{c_{2}}^{2}-{\overset{˜}{\Gamma }}_{c_{1}}^{2}{\overset{˜}{\Gamma }}_{c_{2}}^{2}}\;\right],\sqrt{\eta_{c_{1}}^{2}+\eta_{c_{2}}^{2}-\eta_{c_{1}}^{2}\eta_{c_{2}}^{2}}\;\rangle ) \end{array}$
(iii) $\begin{array}{c} kc_{1}=\left(\langle \left[\sqrt{1-{\left(1-\Lambda_{c_{1}}^{2}\right)}^{k}},\sqrt{1-{\left(1-\Gamma_{c_{1}}^{2}\right)}^{k}}\right],\sqrt{1-{\left(1-\xi_{c_{1}}^{2}\right)}^{k}}\;\rangle ,\langle \left[{\left({\overset{˜}{\Lambda }}_{c_{1}}\right)}^{k},{\left({\overset{˜}{\Gamma }}_{c_{1}}\right)}^{k}\right],{\left(\eta_{c_{1}}\right)}^{k}\rangle \right) \end{array}$,
(iv) $\begin{array}{c} c_{1}^{k}=\left(\langle \left[{\left(\Lambda_{c_{1}}\right)}^{k},{\left(\Gamma_{c_{1}}\right)}^{k}\right],{\left(\xi_{c_{1}}\right)}^{k}\rangle ,\langle \left[\sqrt{1-{\left(1-{\overset{˜}{\Lambda }}_{c_{1}}^{2}\right)}^{k}},\sqrt{1-{\left(1-{\overset{˜}{\Gamma }}_{c_{1}}^{2}\right)}^{k}}\right],\sqrt{1-{\left(1-\eta_{c_{1}}^{2}\right)}^{k}}\;\rangle \right) \end{array}$.

Definition 2.7 [74] Let $c=(\langle A_{c},\xi_{c}\rangle ,\langle {\overset{˜}{A}}_{c},\eta_{c}\rangle )$ be a PCFN, where $A_{c}=[\Lambda_{c},\Gamma_{c}],{\overset{˜}{A}}_{c}=[{\overset{˜}{\Lambda }}_{c},{\overset{˜}{\Gamma }}_{c}]$. Then the score value S(c), and accuracy value H(c) are given by,

$$S(c)={\left(\frac{\Lambda_{c}+\Gamma_{c}-\xi_{c}}{3}\right)}^{2}-{\left(\frac{{\overset{˜}{\Lambda }}_{c}+{\overset{˜}{\Gamma }}_{c}-\eta_{c}}{3}\right)}^{2},\;S(c)\in [-1,1]$$

$$S(c)={\left(\frac{\Lambda_{c}+\Gamma_{c}-\xi_{c}}{3}\right)}^{2}+{\left(\frac{{\overset{˜}{\Lambda }}_{c}+{\overset{˜}{\Gamma }}_{c}-\eta_{c}}{3}\right)}^{2},\;S(c)\in [0,1]$$

Definition 2.8 [74] Let $c_{1}=(\langle A_{c_{1}},\xi_{c_{1}}\rangle ,\langle {\overset{˜}{A}}_{c_{1}},\eta_{c_{1}}\rangle )$ and $c_{2}=(\langle A_{c_{2}},\xi_{c_{2}}\rangle ,\langle {\overset{˜}{A}}_{c_{2}},\eta_{c_{2}}\rangle )$ be two PCFNs, then the following rules hold:

**(R 1)** if $S(c_{1})<S(c_{2})$, then $c_{1}≼c_{2}$
**(R 2)** if $S(c_{1})=S(c_{2})$, then we have,
  **(T 1)** if $H(c_{1})<H(c_{2})$, then $c_{1}≼c_{2}$
  **(T 2)** if $H(c_{1})=H(c_{2})$, then $c_{1}∼c_{2}$.

Definition 2.9 [25] Let $\beta_{j}\;(j=1,2,\ldots ,l)$ be a group of positive reals, and $r=(r_{1},r_{2},\ldots ,r_{l})\in R^{l}$ be a vector parameter, then the MM operator is given as

$${\mathrm{MM}}^{r}(\beta_{1},\beta_{2},\ldots ,\beta_{l})={\left(\frac{1}{l!}\sum_{\theta \in S_{l}}\overset{l}{\prod_{j=1}}\beta_{\theta (j)}^{r_{j}}\right)}^{\frac{1}{\overset{l}{\sum_{j=1}}r_{j}}},$$

here θ(j) represents a permutation in $S_{l}$ of all possible permutations.

Frank [79] introduced the Frank triangular norms, which are expressed as:

Definition 2.10 [79] Let ϑ>1 and $v_{1},v_{2}\in [0,1]$, then Frank *t*-norms and Frank *t*-conorms are expressed as

$$T_{F}(v_{1},v_{2})=\log_{\vartheta }\left(1+\frac{\left(\vartheta^{v_{1}}-1)(\vartheta^{v_{2}}-1\right)}{\vartheta -1}\right),S_{F}(v_{1},v_{2})=1-\log_{\vartheta }\left(1+\frac{\left(\vartheta^{1-v_{1}}-1)(\vartheta^{1-v_{2}}-1\right)}{\vartheta -1}\right),$$

The Frank triangular norms [79] in terms of additive generators is given by Table 1, here f and g can produce *t*-norm $T(v_{1},v_{2})=f^{-1}(f(v_{1})+f(v_{2}))$, and *t*-conorm $S(v_{1},v_{2})=g^{-1}(g(v_{1})+g(v_{2}))$.

**Table 1:** Frank triangular norms with generators.

Operator	Triangular norms	Additive generator
Frank *t*-norm	$T_{F}(v_{1},v_{2})=\log_{\vartheta }\left(1+\frac{\left(\vartheta^{v_{1}}-1)(\vartheta^{v_{2}}-1\right)}{\vartheta -1}\right)$	$f(t)=\ln\left(\frac{\vartheta -1}{\vartheta^{t}-1}\right)$,
Frank *t*-conorm	$S_{F}(v_{1},v_{2})=1-\log_{\vartheta }\left(1+\frac{\left(\vartheta^{1-v_{1}}-1)(\vartheta^{1-v_{2}}-1\right)}{\vartheta -1}\right)$	$g(t)=\ln\left(\frac{\vartheta -1}{\vartheta^{1-t}-1}\right)$,

## 3 Frank operations on PCFNs

This section introduces some Frank operational rules on the PCFNs based on the Frank triangular norms.

Definition 3.1 Let $\beta_{1}=(\langle A_{\beta_{1}},\xi_{\beta_{1}}\rangle ,\langle {\overset{˜}{A}}_{\beta_{1}},\eta_{\beta_{1}}\rangle )$ and $\beta_{2}=(\langle A_{\beta_{2}},\xi_{\beta_{2}}\rangle ,\langle {\overset{˜}{A}}_{\beta_{2}},\eta_{\beta_{2}}\rangle )$ be two PCFNs, where $A_{\beta_{1}}=[\Lambda_{\beta_{1}},\Gamma_{\beta_{1}}],{\overset{˜}{A}}_{\beta_{1}}=[{\overset{˜}{\Lambda }}_{\beta_{1}},{\overset{˜}{\Gamma }}_{\beta_{1}}]$; $A_{\beta_{2}}=[\Lambda_{\beta_{2}},\Gamma_{\beta_{2}}]$, ${\overset{˜}{A}}_{\beta_{2}}=[{\overset{˜}{\Lambda }}_{\beta_{2}},{\overset{˜}{\Gamma }}_{\beta_{2}}]$. Then for scalars k,ϑ>0, the following rules hold:

(i) $$\beta_{1}\;{⊕}_{F}\beta_{2}=(\langle \left[{\left(g^{-1}(g(\Lambda_{\beta_{1}}^{2})+g(\Lambda_{\beta_{2}}^{2}))\right)}^{\frac{1}{2}},{\left(g^{-1}(g(\Gamma_{\beta_{1}}^{2})+g(\Gamma_{\beta_{2}}^{2}))\right)}^{\frac{1}{2}}\right],{\left(g^{-1}(g(\xi_{\beta_{1}}^{2})+g(\xi_{\beta_{2}}^{2}))\right)}^{\frac{1}{2}}\rangle ,\langle \left[{\left(f^{-1}(f({\overset{˜}{\Lambda }}_{\beta_{1}}^{2})+f({\overset{˜}{\Lambda }}_{\beta_{2}}^{2}))\right)}^{\frac{1}{2}},{\left(f^{-1}(f({\overset{˜}{\Gamma }}_{\beta_{1}}^{2})+f({\overset{˜}{\Gamma }}_{\beta_{2}}^{2}))\right)}^{\frac{1}{2}}\right],{\left(f^{-1}(f(\eta_{\beta_{1}}^{2})+f(\eta_{\beta_{2}}^{2}))\right)}^{\frac{1}{2}}\rangle )=(\langle \left[\sqrt{1-\log_{\vartheta }\left(1+\frac{\left(\vartheta^{1-\Lambda_{\beta_{1}}^{2}}-1)(\vartheta^{1-\Lambda_{\beta_{2}}^{2}}-1\right)}{\vartheta -1}\right)},\sqrt{1-\log_{\vartheta }\left(1+\frac{\left(\vartheta^{1-\Gamma_{\beta_{1}}^{2}}-1)(\vartheta^{1-\Gamma_{\beta_{2}}^{2}}-1\right)}{\vartheta -1}\right)}\;\right],\sqrt{1-\log_{\vartheta }\left(1+\frac{\left(\vartheta^{1-\xi_{\beta_{1}}^{2}}-1)(\vartheta^{1-\xi_{\beta_{2}}^{2}}-1\right)}{\vartheta -1}\right)}\;\rangle ,\langle \left[\sqrt{\log_{\vartheta }\left(1+\frac{\left(\vartheta^{{\overset{˜}{\Lambda }}_{\beta_{1}}^{2}}-1)(\vartheta^{{\overset{˜}{\Lambda }}_{\beta_{2}}^{2}}-1\right)}{\vartheta -1}\right)},\sqrt{\log_{\vartheta }\left(1+\frac{\left(\vartheta^{{\overset{˜}{\Gamma }}_{\beta_{1}}^{2}}-1)(\vartheta^{{\overset{˜}{\Gamma }}_{\beta_{2}}^{2}}-1\right)}{\vartheta -1}\right)}\;\right],\sqrt{\log_{\vartheta }\left(1+\frac{\left(\vartheta^{\eta_{\beta_{1}}^{2}}-1)(\vartheta^{\eta_{\beta_{2}}^{2}}-1\right)}{\vartheta -1}\right)}\;\rangle )$$
(ii) $$\beta_{1}\;{⊗}_{F}\beta_{2}=(\langle \left[{\left(f^{-1}(f(\Lambda_{\beta_{1}}^{2})+f(\Lambda_{\beta_{2}}^{2}))\right)}^{\frac{1}{2}},{\left(f^{-1}(f(\Gamma_{\beta_{1}}^{2})+f(\Gamma_{\beta_{2}}^{2}))\right)}^{\frac{1}{2}}\right],{\left(f^{-1}(f(\xi_{\beta_{1}}^{2})+f(\xi_{\beta_{2}}^{2}))\right)}^{\frac{1}{2}}\rangle ,\langle \left[{\left(g^{-1}(g({\overset{˜}{\Lambda }}_{\beta_{1}}^{2})+g({\overset{˜}{\Lambda }}_{\beta_{2}}^{2}))\right)}^{\frac{1}{2}},{\left(g^{-1}(g({\overset{˜}{\Gamma }}_{\beta_{1}}^{2})+g({\overset{˜}{\Gamma }}_{\beta_{2}}^{2}))\right)}^{\frac{1}{2}}\right],{\left(g^{-1}(g(\eta_{\beta_{1}}^{2})+g(\eta_{\beta_{2}}^{2}))\right)}^{\frac{1}{2}}\rangle )=(\langle \left[\sqrt{\log_{\vartheta }\left(1+\frac{\left(\vartheta^{\Lambda_{\beta_{1}}^{2}}-1)(\vartheta^{\Lambda_{\beta_{2}}^{2}}-1\right)}{\vartheta -1}\right)},\sqrt{\log_{\vartheta }\left(1+\frac{\left(\vartheta^{\Gamma_{\beta_{1}}^{2}}-1)(\vartheta^{\Gamma_{\beta_{2}}^{2}}-1\right)}{\vartheta -1}\right)}\;\right],\sqrt{\log_{\vartheta }\left(1+\frac{\left(\vartheta^{\xi_{\beta_{1}}^{2}}-1)(\vartheta^{\xi_{\beta_{2}}^{2}}-1\right)}{\vartheta -1}\right)}\;\rangle ,\langle \left[\sqrt{1-\log_{\vartheta }\left(1+\frac{\left(\vartheta^{1-{\overset{˜}{\Lambda }}_{\beta_{1}}^{2}}-1)(\vartheta^{1-{\overset{˜}{\Lambda }}_{\beta_{2}}^{2}}-1\right)}{\vartheta -1}\right)},\sqrt{1-\log_{\vartheta }\left(1+\frac{\left(\vartheta^{1-{\overset{˜}{\Gamma }}_{\beta_{1}}^{2}}-1)(\vartheta^{1-{\overset{˜}{\Gamma }}_{\beta_{2}}^{2}}-1\right)}{\vartheta -1}\right)}\;\right],\sqrt{1-\log_{\vartheta }\left(1+\frac{\left(\vartheta^{1-\eta_{\beta_{1}}^{2}}-1)(\vartheta^{1-\eta_{\beta_{2}}^{2}}-1\right)}{\vartheta -1}\right)}\;\rangle )$$
(iii) $$k\beta_{1}=(\langle \left[{\left(g^{-1}(kg(\Lambda_{\beta_{1}}^{2}))\right)}^{\frac{1}{2}},{\left(g^{-1}(kg(\Gamma_{\beta_{1}}^{2}))\right)}^{\frac{1}{2}}\right],{\left(g^{-1}(kg(\xi_{\beta_{1}}^{2}))\right)}^{\frac{1}{2}}\rangle ,\langle \left[{\left(f^{-1}(kf({\overset{˜}{\Lambda }}_{\beta_{1}}^{2}))\right)}^{\frac{1}{2}},{\left(f^{-1}(kf({\overset{˜}{\Gamma }}_{\beta_{1}}^{2}))\right)}^{\frac{1}{2}}\right],{\left(f^{-1}(kf(\eta_{\beta_{1}}^{2}))\right)}^{\frac{1}{2}}\rangle )=(\langle \left[\sqrt{1-\log_{\vartheta }\left(1+\frac{{\left(\vartheta^{1-\Lambda_{\beta_{1}}^{2}}-1\right)}^{k}}{{\left(\vartheta -1\right)}^{k-1}}\right)},\sqrt{1-\log_{\vartheta }\left(1+\frac{{\left(\vartheta^{1-\Gamma_{\beta_{1}}^{2}}-1\right)}^{k}}{{\left(\vartheta -1\right)}^{k-1}}\right)}\;\right],\sqrt{1-\log_{\vartheta }\left(1+\frac{{\left(\vartheta^{1-\xi_{\beta_{1}}^{2}}-1\right)}^{k}}{{\left(\vartheta -1\right)}^{k-1}}\right)}\;\rangle ,\langle \left[\sqrt{\log_{\vartheta }\left(1+\frac{{\left(\vartheta^{{\overset{˜}{\Lambda }}_{\beta_{1}}^{2}}-1\right)}^{k}}{{\left(\vartheta -1\right)}^{k-1}}\right)},\sqrt{\log_{\vartheta }\left(1+\frac{{\left(\vartheta^{{\overset{˜}{\Gamma }}_{\beta_{1}}^{2}}-1\right)}^{k}}{{\left(\vartheta -1\right)}^{k-1}}\right)}\;\right],\sqrt{\log_{\vartheta }\left(1+\frac{{\left(\vartheta^{\xi_{\beta_{1}}^{2}}-1\right)}^{k}}{{\left(\vartheta -1\right)}^{k-1}}\right)}\;\rangle )$$
(iv) $$\beta_{1}^{k}=(\langle \left[{\left(f^{-1}(kf(\Lambda_{\beta_{1}}^{2}))\right)}^{\frac{1}{2}},{\left(f^{-1}(f(\Gamma_{\beta_{1}}^{2}))\right)}^{\frac{1}{2}}\right],{\left(f^{-1}(kf(\xi_{\beta_{1}}^{2}))\right)}^{\frac{1}{2}}\rangle ,\langle \left[{\left(g^{-1}(kg({\overset{˜}{\Lambda }}_{\beta_{1}}^{2}))\right)}^{\frac{1}{2}},{\left(g^{-1}(kg({\overset{˜}{\Gamma }}_{\beta_{1}}^{2}))\right)}^{\frac{1}{2}}\right],{\left(g^{-1}(kg(\eta_{\beta_{1}}^{2}))\right)}^{\frac{1}{2}}\rangle )=(\langle \left[\sqrt{\log_{\vartheta }\left(1+\frac{{\left(\vartheta^{\Lambda_{\beta_{1}}^{2}}-1\right)}^{k}}{{\left(\vartheta -1\right)}^{k-1}}\right)},\sqrt{\log_{\vartheta }\left(1+\frac{{\left(\vartheta^{\Gamma_{\beta_{1}}^{2}}-1\right)}^{k}}{{\left(\vartheta -1\right)}^{k-1}}\right)}\;\right],\sqrt{\log_{\vartheta }\left(1+\frac{{\left(\vartheta^{\xi_{\beta_{1}}^{2}}-1\right)}^{k}}{{\left(\vartheta -1\right)}^{k-1}}\right)}\;\rangle ,\langle \left[\sqrt{1-\log_{\vartheta }\left(1+\frac{{\left(\vartheta^{1-{\overset{˜}{\Lambda }}_{\beta_{1}}^{2}}-1\right)}^{k}}{{\left(\vartheta -1\right)}^{k-1}}\right)},\sqrt{1-\log_{\vartheta }\left(1+\frac{{\left(\vartheta^{1-{\overset{˜}{\Gamma }}_{\beta_{1}}^{2}}-1\right)}^{k}}{{\left(\vartheta -1\right)}^{k-1}}\right)}\;\right],\sqrt{1-\log_{\vartheta }\left(1+\frac{{\left(\vartheta^{1-\xi_{\beta_{1}}^{2}}-1\right)}^{k}}{{\left(\vartheta -1\right)}^{k-1}}\right)}\;\rangle )$$

Definition 3.2 Let $\beta ,\beta_{1}$ and $\beta_{2}$ be three PCFNs, $k,k_{1},k_{1}>0$ are scalars and if ${⨁}_{F},{⨂}_{F}$ respectively represent the Frank sum and product, then the following hold:

(i) $\beta_{1}{⨁}_{F}\beta_{2}=\beta_{2}{⨁}_{F}\beta_{1}$
(ii) $\beta_{1}{⨂}_{F}\beta_{2}=\beta_{2}{⨂}_{F}\beta_{1}$
(iii) $k(\beta_{1}{⨁}_{F}\beta_{2})=k\beta_{1}{⨁}_{F}k\beta_{2}$
(iv) ${\left(\beta_{1}{⨂}_{F}\beta_{2}\right)}^{k}=\beta_{1}^{k}{⨂}_{F}\beta_{2}^{k}$
(v) $(k_{1}+k_{2})\beta =k_{1}\beta {⨁}_{F}k_{2}\beta$
(vi) $\beta^{\left(k_{1}+k_{2}\right)}=\beta^{k_{1}}{⨂}_{F}\beta^{k_{2}}$
(vii) ${\left(\beta^{k_{1}}\right)}^{k_{2}}=\beta^{k_{1}k_{2}}$

## 4 PCFFMM and PCFFGMM operators

This section proposes the PCFFMM and PCFFGMM operators and analyses their important features.

Definition 4.1 Suppose $\beta_{j}=\left\{\left(\langle [\Lambda_{\beta_{j}},\Gamma_{\beta_{j}}],\xi_{\beta_{j}}\rangle ,\langle [{\overset{˜}{\Lambda }}_{\beta_{j}},{\overset{˜}{\Gamma }}_{\beta_{j}}],\eta_{\beta_{j}}\rangle \right)|1⩽j⩽n\right\}$ be a group of PCFNs on V, and $r=(r_{1},r_{2},\ldots ,r_{n})\in R^{n}$ be a vector of parameter. Then the Pythagorean cubic fuzzy Frank Muirhead mean (PCFFMM) operator is given as

$$\mathrm{PCFFMM}(\beta_{1},\beta_{2},\ldots ,\beta_{n})={\left(\frac{1}{n!}\left(\underset{\theta \in S_{n}}{{⨁}_{F}}\left(\overset{n}{\underset{j=1}{{⨂}_{F}}}\beta_{\theta (j)}^{r_{j}}\right)\right)\right)}^{\frac{1}{\overset{n}{\sum_{j=1}}r_{j}}},$$

here θ(j) denotes a permutation in $S_{n}$ of all permutations of {1,2,…,n}. Also, here ${⨁}_{F}$ and ${⨂}_{F}$ respectively denotes the Frank sum and the Frank product of PCFNs given by (i) and (ii) in Definition 3.1.

Theorem 4.1 *Let*$\beta_{j}=\left\{\left(\langle [\Lambda_{\beta_{j}},\Gamma_{\beta_{j}}],\xi_{\beta_{j}}\rangle ,\langle [{\overset{˜}{\Lambda }}_{\beta_{j}},{\overset{˜}{\Gamma }}_{\beta_{j}}],\eta_{\beta_{j}}\rangle \right)|1⩽j⩽n\right\}$*be a set of PCFNs on*V*, then* PCFFMM *can be given as*

(1) $$\mathrm{PCFFMM}(\beta_{1},\beta_{2},\ldots ,\beta_{n})=(\langle [{\left(f^{-1}\left(\frac{1}{\overset{n}{\sum_{j=1}}r_{j}}\;f\left(g^{-1}\left(\frac{1}{n!}\sum_{\theta \in S_{n}}g\left(f^{-1}\left(\overset{n}{\sum_{j=1}}r_{j}f\left(\Lambda_{\beta_{\theta (j)}}^{2}\right)\right)\right)\right)\right)\right)\right)}^{1/2},{\left(f^{-1}\left(\frac{1}{\overset{n}{\sum_{j=1}}r_{j}}\;f\left(g^{-1}\left(\frac{1}{n!}\sum_{\theta \in S_{n}}g\left(f^{-1}\left(\overset{n}{\sum_{j=1}}r_{j}f\left(\Gamma_{\beta_{\theta (j)}}^{2}\right)\right)\right)\right)\right)\right)\right)}^{1/2}],{\left(f^{-1}\left(\frac{1}{\overset{n}{\sum_{j=1}}r_{j}}\;f\left(g^{-1}\left(\frac{1}{n!}\sum_{\theta \in S_{n}}g\left(f^{-1}\left(\overset{n}{\sum_{j=1}}r_{j}f\left(\xi_{\beta_{\theta (j)}}^{2}\right)\right)\right)\right)\right)\right)\right)}^{1/2}\rangle \langle [{\left(g^{-1}\left(\frac{1}{\overset{n}{\sum_{j=1}}r_{j}}\;g\left(f^{-1}\left(\frac{1}{n!}\sum_{\theta \in S_{n}}f\left(g^{-1}\left(\overset{n}{\sum_{j=1}}r_{j}g\left({\overset{˜}{\Lambda }}_{\beta_{\theta (j)}}^{2}\right)\right)\right)\right)\right)\right)\right)}^{1/2},{\left(g^{-1}\left(\frac{1}{\overset{n}{\sum_{j=1}}r_{j}}\;g\left(f^{-1}\left(\frac{1}{n!}\sum_{\theta \in S_{n}}f\left(g^{-1}\left(\overset{n}{\sum_{j=1}}r_{j}g\left({\overset{˜}{\Gamma }}_{\beta_{\theta (j)}}^{2}\right)\right)\right)\right)\right)\right)\right)}^{1/2}],{\left(g^{-1}\left(\frac{1}{\overset{n}{\sum_{j=1}}r_{j}}\;g\left(f^{-1}\left(\frac{1}{n!}\sum_{\theta \in S_{n}}f\left(g^{-1}\left(\overset{n}{\sum_{j=1}}r_{j}g\left(\eta_{\beta_{\theta (j)}}^{2}\right)\right)\right)\right)\right)\right)\right)}^{1/2}\rangle )$$

*Equation* (1) *denotes the* PCFFMM *operator by means of generators* f *and* g *of Frank triangular norms* *[79]**.*

*Where,*

$$\begin{array}{ccc} f(v)=\ln\left(\frac{\vartheta -1}{\vartheta^{v}-1}\right)\; & ,\; & g(v)=\ln\left(\frac{\vartheta -1}{\vartheta^{1-v}-1}\right)\\ f^{-1}(v)=\log_{\vartheta }\left(1+\frac{\vartheta -1}{e^{v}}\right)\; & ,\; & g^{-1}(v)=1-\log_{\vartheta }\left(1+\frac{\vartheta -1}{e^{v}}\right). \end{array}$$

Theorem 4.2 *Let*$\beta_{j}=\left\{\left(\langle [\Lambda_{\beta_{j}},\Gamma_{\beta_{j}}],\xi_{\beta_{j}}\rangle ,\langle [{\overset{˜}{\Lambda }}_{\beta_{j}},{\overset{˜}{\Gamma }}_{\beta_{j}}],\eta_{\beta_{j}}\rangle \right)|1⩽j⩽n\right\}$*be a set of PCFNs on*V*, and ϑ a parameter, then the collective value derived by using* PCFFMM *is also a PCFN, which is presented as:*

$$\mathrm{PCFFMM}(\beta_{1},\beta_{2},\ldots ,\beta_{n})=(\langle [\sqrt{\log_{\vartheta }\left(1+(\vartheta -1){\left(\frac{1-\prod_{\theta (j)\in S_{n}}{\left(\frac{1-\overset{n}{\prod_{j=1}}\zeta_{\theta (j)}}{1+(\vartheta -1)\overset{n}{\prod_{j=1}}\zeta_{\theta (j)}}\right)}^{\frac{1}{n!}}}{1+(\vartheta -1)\prod_{\theta (j)\in S_{n}}{\left(\frac{1-\overset{n}{\prod_{j=1}}\zeta_{\theta (j)}}{1+(\vartheta -1)\overset{n}{\prod_{j=1}}\zeta_{\theta (j)}}\right)}^{\frac{1}{n!}}}\right)}^{\frac{1}{\overset{n}{\sum_{j=1}}r_{j}}}\right)},\sqrt{\log_{\vartheta }\left(1+(\vartheta -1){\left(\frac{1-\prod_{\theta (j)\in S_{n}}{\left(\frac{1-\overset{n}{\prod_{j=1}}\tau_{\theta (j)}}{1+(\vartheta -1)\overset{n}{\prod_{j=1}}\tau_{\theta (j)}}\right)}^{\frac{1}{n!}}}{1+(\vartheta -1)\prod_{\theta (j)\in S_{n}}{\left(\frac{1-\overset{n}{\prod_{j=1}}\tau_{\theta (j)}}{1+(\vartheta -1)\overset{n}{\prod_{j=1}}\tau_{\theta (j)}}\right)}^{\frac{1}{n!}}}\right)}^{\frac{1}{\overset{n}{\sum_{j=1}}r_{j}}}\right)}\;],\sqrt{\log_{\vartheta }\left(1+(\vartheta -1){\left(\frac{1-\prod_{\theta (j)\in S_{n}}{\left(\frac{1-\overset{n}{\prod_{j=1}}\sigma_{\theta (j)}}{1+(\vartheta -1)\overset{n}{\prod_{j=1}}\sigma_{\theta (j)}}\right)}^{\frac{1}{n!}}}{1+(\vartheta -1)\prod_{\theta (j)\in S_{n}}{\left(\frac{1-\overset{n}{\prod_{j=1}}\sigma_{\theta (j)}}{1+(\vartheta -1)\overset{n}{\prod_{j=1}}\sigma_{\theta (j)}}\right)}^{\frac{1}{n!}}}\right)}^{\frac{1}{\overset{n}{\sum_{j=1}}r_{j}}}\right)}\rangle ,\langle [\sqrt{1-\log_{\vartheta }\left(1+(\vartheta -1){\left(\frac{1-\prod_{\theta (j)\in S_{n}}{\left(\frac{1-\overset{n}{\prod_{j=1}}{\overset{˜}{\zeta }}_{\theta (j)}}{1+(\vartheta -1)\overset{n}{\prod_{j=1}}{\overset{˜}{\zeta }}_{\theta (j)}}\right)}^{\frac{1}{n!}}}{1+(\vartheta -1)\prod_{\theta (j)\in S_{n}}{\left(\frac{1-\overset{n}{\prod_{j=1}}{\overset{˜}{\zeta }}_{\theta (j)}}{1+(\vartheta -1)\overset{n}{\prod_{j=1}}{\overset{˜}{\zeta }}_{\theta (j)}}\right)}^{\frac{1}{n!}}}\right)}^{\frac{1}{\overset{n}{\sum_{j=1}}r_{j}}}\right)},\sqrt{1-\log_{\vartheta }\left(1+(\vartheta -1){\left(\frac{1-\prod_{\theta (j)\in S_{n}}{\left(\frac{1-\overset{n}{\prod_{j=1}}{\overset{˜}{\tau }}_{\theta (j)}}{1+(\vartheta -1)\overset{n}{\prod_{j=1}}{\overset{˜}{\tau }}_{\theta (j)}}\right)}^{\frac{1}{n!}}}{1+(\vartheta -1)\prod_{\theta (j)\in S_{n}}{\left(\frac{1-\overset{n}{\prod_{j=1}}{\overset{˜}{\tau }}_{\theta (j)}}{1+(\vartheta -1)\overset{n}{\prod_{j=1}}{\overset{˜}{\tau }}_{\theta (j)}}\right)}^{\frac{1}{n!}}}\right)}^{\frac{1}{\overset{n}{\sum_{j=1}}r_{j}}}\right)}\;],\sqrt{1-\log_{\vartheta }\left(1+(\vartheta -1){\left(\frac{1-\prod_{\theta (j)\in S_{n}}{\left(\frac{1-\overset{n}{\prod_{j=1}}\rho_{\theta (j)}}{1+(\vartheta -1)\overset{n}{\prod_{j=1}}\rho_{\theta (j)}}\right)}^{\frac{1}{n!}}}{1+(\vartheta -1)\prod_{\theta (j)\in S_{n}}{\left(\frac{1-\overset{n}{\prod_{j=1}}\rho_{\theta (j)}}{1+(\vartheta -1)\overset{n}{\prod_{j=1}}\rho_{\theta (j)}}\right)}^{\frac{1}{n!}}}\right)}^{\frac{1}{\overset{n}{\sum_{j=1}}r_{j}}}\right)}\rangle )$$

*where*

$$\begin{array}{ccc} \zeta_{\theta (j)}={\left(\frac{\vartheta^{\Lambda_{\beta_{\theta (j)}}^{2}}-1}{\vartheta -1}\right)}^{r_{j}},\; & \tau_{\theta (j)}={\left(\frac{\vartheta^{\Gamma_{\beta_{\theta (j)}}^{2}}-1}{\vartheta -1}\right)}^{r_{j}},\; & \sigma_{\theta (j)}={\left(\frac{\vartheta^{\xi_{\beta_{\theta (j)}}^{2}}-1}{\vartheta -1}\right)}^{r_{j}}\\ \; & \; & \\ {\overset{˜}{\zeta }}_{\theta (j)}={\left(\frac{\vartheta^{\left(1-{\overset{˜}{\Lambda }}_{\beta_{\theta (j)}}^{2}\right)}-1}{\vartheta -1}\right)}^{r_{j}},\; & {\overset{˜}{\tau }}_{\theta (j)}={\left(\frac{\vartheta^{\left(1-{\overset{˜}{\Gamma }}_{\beta_{\theta (j)}}^{2}\right)}-1}{\vartheta -1}\right)}^{r_{j}},\; & \rho_{\theta (j)}={\left(\frac{\vartheta^{\left(1-\eta_{\beta_{\theta (j)}}^{2}\right)}-1}{\vartheta -1}\right)}^{r_{j}}. \end{array}$$

Definition 4.2 Let $\beta_{j}=\left\{\left(\langle [\Lambda_{\beta_{j}},\Gamma_{\beta_{j}}],\xi_{\beta_{j}}\rangle ,\langle [{\overset{˜}{\Lambda }}_{\beta_{j}},{\overset{˜}{\Gamma }}_{\beta_{j}}],\eta_{\beta_{j}}\rangle \right)|1⩽j⩽n\right\}$ be a set of PCFNs on V, and $r=(r_{1},r_{2},\ldots ,r_{n})\in R^{n}$ be a vector of parameter. Then the Pythagorean cubic fuzzy Frank geometric MM (PCFFGMM) is given as:

$$\mathrm{PCFFGMM}(\beta_{1},\beta_{2},\ldots ,\beta_{n})=\frac{1}{\overset{n}{\sum_{j=1}}r_{j}}\left(\underset{\theta \in S_{n}}{{⨂}_{F}}{\left(\overset{n}{\underset{j=1}{{⨁}_{F}}}\left(r_{j}\beta_{\theta (j)}\right)\right)}^{\frac{1}{n!}}\right),$$

here ${⨁}_{F}$ and ${⨂}_{F}$ respectively denotes the Frank sum and the Frank product of PCFNs given by (i) and (ii) in Definition 3.1.

Theorem 4.3 *Let*$\beta_{j}=\left\{\left(\langle [\Lambda_{\beta_{j}},\Gamma_{\beta_{j}}],\xi_{\beta_{j}}\rangle ,\langle [{\overset{˜}{\Lambda }}_{\beta_{j}},{\overset{˜}{\Gamma }}_{\beta_{j}}],\eta_{\beta_{j}}\rangle \right)|1⩽j⩽n\right\}$*be a group of PCFNs on*V*, then the* PCFFGMM *is given by*

(2) $$\mathrm{PCFFGMM}(\beta_{1},\beta_{2},\ldots ,\beta_{n})=(\langle [{\left(g^{-1}\left(\frac{1}{\overset{n}{\sum_{j=1}}r_{j}}\;g\left(f^{-1}\left(\frac{1}{n!}\sum_{\theta \in S_{n}}f\left(g^{-1}\left(\overset{n}{\sum_{j=1}}r_{j}g\left(\Lambda_{\beta_{\theta (j)}}^{2}\right)\right)\right)\right)\right)\right)\right)}^{1/2},{\left(g^{-1}\left(\frac{1}{\overset{n}{\sum_{j=1}}r_{j}}\;g\left(f^{-1}\left(\frac{1}{n!}\sum_{\theta \in S_{n}}f\left(g^{-1}\left(\overset{n}{\sum_{j=1}}r_{j}g\left(\Gamma_{\beta_{\theta (j)}}^{2}\right)\right)\right)\right)\right)\right)\right)}^{1/2}],{\left(g^{-1}\left(\frac{1}{\overset{n}{\sum_{j=1}}r_{j}}\;g\left(f^{-1}\left(\frac{1}{n!}\sum_{\theta \in S_{n}}f\left(g^{-1}\left(\overset{n}{\sum_{j=1}}r_{j}g\left(\xi_{\beta_{\theta (j)}}^{2}\right)\right)\right)\right)\right)\right)\right)}^{1/2}\rangle \langle [{\left(f^{-1}\left(\frac{1}{\overset{n}{\sum_{j=1}}r_{j}}\;f\left(g^{-1}\left(\frac{1}{n!}\sum_{\theta \in S_{n}}g\left(f^{-1}\left(\overset{n}{\sum_{j=1}}r_{j}f\left({\overset{˜}{\Lambda }}_{\beta_{\theta (j)}}^{2}\right)\right)\right)\right)\right)\right)\right)}^{1/2},{\left(f^{-1}\left(\frac{1}{\overset{n}{\sum_{j=1}}r_{j}}\;f\left(g^{-1}\left(\frac{1}{n!}\sum_{\theta \in S_{n}}g\left(f^{-1}\left(\overset{n}{\sum_{j=1}}r_{j}f\left({\overset{˜}{\Gamma }}_{\beta_{\theta (j)}}^{2}\right)\right)\right)\right)\right)\right)\right)}^{1/2}],{\left(f^{-1}\left(\frac{1}{\overset{n}{\sum_{j=1}}r_{j}}\;f\left(g^{-1}\left(\frac{1}{n!}\sum_{\theta \in S_{n}}g\left(f^{-1}\left(\overset{n}{\sum_{j=1}}r_{j}f\left(\eta_{\beta_{\theta (j)}}^{2}\right)\right)\right)\right)\right)\right)\right)}^{1/2}\rangle )$$

*Equation* (2) *expresses the* PCFFGMM *operator in terms of the additive generators* f *and* g *of Frank triangular norms* *[79]**.*

Theorem 4.4 *Let*$\beta_{j}=\left\{\left(\langle [\Lambda_{\beta_{j}},\Gamma_{\beta_{j}}],\xi_{\beta_{j}}\rangle ,\langle [{\overset{˜}{\Lambda }}_{\beta_{j}},{\overset{˜}{\Gamma }}_{\beta_{j}}],\eta_{\beta_{j}}\rangle \right)|1⩽j⩽n\right\}$*be a set of PCFNs on*V*, and ϑ be a parameter, then the aggregated result obtained by using* PCFFGMM *operator is also a PCFN, given as follows:*

$$\mathrm{PCFFGMM}(\beta_{1},\beta_{2},\ldots ,\beta_{n})=(\langle [\sqrt{1-\log_{\vartheta }\left(1+(\vartheta -1){\left(\frac{1-\prod_{\theta (j)\in S_{n}}{\left(\frac{1-\overset{n}{\prod_{j=1}}\zeta_{\theta (j)}^{⁎}}{1+(\vartheta -1)\overset{n}{\prod_{j=1}}\zeta_{\theta (j)}^{⁎}}\right)}^{\frac{1}{n!}}}{1+(\vartheta -1)\prod_{\theta (j)\in S_{n}}{\left(\frac{1-\overset{n}{\prod_{j=1}}\zeta_{\theta (j)}^{⁎}}{1+(\vartheta -1)\overset{n}{\prod_{j=1}}\zeta_{\theta (j)}^{⁎}}\right)}^{\frac{1}{n!}}}\right)}^{\frac{1}{\overset{n}{\sum_{j=1}}r_{j}}}\right)},\sqrt{1-\log_{\vartheta }\left(1+(\vartheta -1){\left(\frac{1-\prod_{\theta (j)\in S_{n}}{\left(\frac{1-\overset{n}{\prod_{j=1}}\tau_{\theta (j)}^{⁎}}{1+(\vartheta -1)\overset{n}{\prod_{j=1}}\tau_{\theta (j)}^{⁎}}\right)}^{\frac{1}{n!}}}{1+(\vartheta -1)\prod_{\theta (j)\in S_{n}}{\left(\frac{1-\overset{n}{\prod_{j=1}}\tau_{\theta (j)}^{⁎}}{1+(\vartheta -1)\overset{n}{\prod_{j=1}}\tau_{\theta (j)}^{⁎}}\right)}^{\frac{1}{n!}}}\right)}^{\frac{1}{\overset{n}{\sum_{j=1}}r_{j}}}\right)}\;],\sqrt{1-\log_{\vartheta }\left(1+(\vartheta -1){\left(\frac{1-\prod_{\theta (j)\in S_{n}}{\left(\frac{1-\overset{n}{\prod_{j=1}}\sigma_{\theta (j)}^{⁎}}{1+(\vartheta -1)\overset{n}{\prod_{j=1}}\sigma_{\theta (j)}^{⁎}}\right)}^{\frac{1}{n!}}}{1+(\vartheta -1)\prod_{\theta (j)\in S_{n}}{\left(\frac{1-\overset{n}{\prod_{j=1}}\sigma_{\theta (j)}^{⁎}}{1+(\vartheta -1)\overset{n}{\prod_{j=1}}\sigma_{\theta (j)}^{⁎}}\right)}^{\frac{1}{n!}}}\right)}^{\frac{1}{\overset{n}{\sum_{j=1}}r_{j}}}\right)}\rangle ,\langle [\sqrt{\log_{\vartheta }\left(1+(\vartheta -1){\left(\frac{1-\prod_{\theta (j)\in S_{n}}{\left(\frac{1-\overset{n}{\prod_{j=1}}{\overset{˜}{\zeta }}_{\theta (j)}^{⁎}}{1+(\vartheta -1)\overset{n}{\prod_{j=1}}{\overset{˜}{\zeta }}_{\theta (j)}^{⁎}}\right)}^{\frac{1}{n!}}}{1+(\vartheta -1)\prod_{\theta (j)\in S_{n}}{\left(\frac{1-\overset{n}{\prod_{j=1}}{\overset{˜}{\zeta }}_{\theta (j)}^{⁎}}{1+(\vartheta -1)\overset{n}{\prod_{j=1}}{\overset{˜}{\zeta }}_{\theta (j)}^{⁎}}\right)}^{\frac{1}{n!}}}\right)}^{\frac{1}{\overset{n}{\sum_{j=1}}r_{j}}}\right)},\sqrt{\log_{\vartheta }\left(1+(\vartheta -1){\left(\frac{1-\prod_{\theta (j)\in S_{n}}{\left(\frac{1-\overset{n}{\prod_{j=1}}{\overset{˜}{\tau }}_{\theta (j)}^{⁎}}{1+(\vartheta -1)\overset{n}{\prod_{j=1}}{\overset{˜}{\tau }}_{\theta (j)}^{⁎}}\right)}^{\frac{1}{n!}}}{1+(\vartheta -1)\prod_{\theta (j)\in S_{n}}{\left(\frac{1-\overset{n}{\prod_{j=1}}{\overset{˜}{\tau }}_{\theta (j)}^{⁎}}{1+(\vartheta -1)\overset{n}{\prod_{j=1}}{\overset{˜}{\tau }}_{\theta (j)}^{⁎}}\right)}^{\frac{1}{n!}}}\right)}^{\frac{1}{\overset{n}{\sum_{j=1}}r_{j}}}\right)}\;],\sqrt{1-\log_{\vartheta }\left(1+(\vartheta -1){\left(\frac{1-\prod_{\theta (j)\in S_{n}}{\left(\frac{1-\overset{n}{\prod_{j=1}}\rho_{\theta (j)}^{⁎}}{1+(\vartheta -1)\overset{n}{\prod_{j=1}}\rho_{\theta (j)}^{⁎}}\right)}^{\frac{1}{n!}}}{1+(\vartheta -1)\prod_{\theta (j)\in S_{n}}{\left(\frac{1-\overset{n}{\prod_{j=1}}\rho_{\theta (j)}^{⁎}}{1+(\vartheta -1)\overset{n}{\prod_{j=1}}\rho_{\theta (j)}^{⁎}}\right)}^{\frac{1}{n!}}}\right)}^{\frac{1}{\overset{n}{\sum_{j=1}}r_{j}}}\right)}\rangle )$$

*where*

$$\begin{array}{ccc} \zeta_{\theta (j)}^{⁎}={\left(\frac{\vartheta^{\left(1-\Lambda_{\beta_{\theta (j)}}^{2}\right)}-1}{\vartheta -1}\right)}^{r_{j}},\; & \tau_{\theta (j)}^{⁎}={\left(\frac{\vartheta^{\left(1-\Gamma_{\beta_{\theta (j)}}^{2}\right)}-1}{\vartheta -1}\right)}^{r_{j}},\; & \sigma_{\theta (j)}^{⁎}={\left(\frac{\vartheta^{\left(\xi_{\beta_{\theta (j)}}^{2}\right)}-1}{\vartheta -1}\right)}^{r_{j}}\\ \; & \; & \\ {\overset{˜}{\zeta }}_{\theta (j)}^{⁎}={\left(\frac{\vartheta^{{\overset{˜}{\Lambda }}_{\beta_{\theta (j)}}^{2}}-1}{\vartheta -1}\right)}^{r_{j}},\; & {\overset{˜}{\tau }}_{\theta (j)}^{⁎}={\left(\frac{\vartheta^{{\overset{˜}{\Gamma }}_{\beta_{\theta (j)}}^{2}}-1}{\vartheta -1}\right)}^{r_{j}},\; & \rho_{\theta (j)}^{⁎}={\left(\frac{\vartheta^{\eta_{\beta_{\theta (j)}}^{2}}-1}{\vartheta -1}\right)}^{r_{j}}. \end{array}$$

Some particular instances of PCFFMM and PCFFGMM are described as follows:

Corollary 4.1 *If the vector parameter*$r=(\overset{l}{\overset{︷}{1,1,\ldots ,1}},\underset{n-l}{\underset{︸}{0,0,\ldots ,0}})$*then the* PCFFMM *reduces to a novel Pythagorean cubic fuzzy Frank MSM* (PCFFMSM)*, which is expressed as:*

$$\mathrm{PCFFMSM}(\beta_{1},\beta_{2},\ldots ,\beta_{n})={\left(\frac{l!(n-l)!}{n!}\left(\underset{1\leq j_{1}\leq j_{2}\leq ,\ldots ,\leq j_{l}\leq n}{{⨁}_{F}}\left(\overset{l}{\underset{t=1}{{⨂}_{F}}}\beta_{j_{t}}\right)\right)\right)}^{\frac{1}{l}},$$

*where,* $\left(j_{1},j_{2},\ldots ,j_{l}\right)$ *sweep over l-tuple combinations of* {1,2,…,n}*.*

*Then,*

$$\mathrm{PCFFMSM}(\beta_{1},\beta_{2},\ldots ,\beta_{n})=(\langle [\sqrt{\log_{\vartheta }\left(1+(\vartheta -1){\left(\frac{1-\prod_{1\leq t_{1}\leq t_{2}\ldots ,\leq j_{l}\leq n}{\left(\frac{1-\overset{l}{\prod_{t=1}}\zeta_{j_{t}}}{1+(\vartheta -1)\overset{l}{\prod_{t=1}}\zeta_{j_{t}}}\right)}^{\frac{l!(n-l)!}{n!}}}{1+(\vartheta -1)\prod_{1\leq t_{1}\leq t_{2}\ldots ,\leq j_{l}\leq n}{\left(\frac{1-\overset{l}{\prod_{t=1}}\zeta_{j_{t}}}{1+(\vartheta -1)\overset{l}{\prod_{t=1}}\zeta_{j_{t}}}\right)}^{\frac{l!(n-l)!}{n!}}}\right)}^{\frac{1}{l}}\right)},\sqrt{\log_{\vartheta }\left(1+(\vartheta -1){\left(\frac{1-\prod_{1\leq t_{1}\leq t_{2}\ldots ,\leq j_{l}\leq n}{\left(\frac{1-\overset{l}{\prod_{t=1}}\tau_{j_{t}}}{1+(\vartheta -1)\overset{l}{\prod_{t=1}}\tau_{j_{t}}}\right)}^{\frac{l!(n-l)!}{n!}}}{1+(\vartheta -1)\prod_{1\leq t_{1}\leq t_{2}\ldots ,\leq j_{l}\leq n}{\left(\frac{1-\overset{l}{\prod_{t=1}}\tau_{j_{t}}}{1+(\vartheta -1)\overset{k}{\prod_{t=1}}\tau_{j_{t}}}\right)}^{\frac{l!(n-l)!}{n!}}}\right)}^{\frac{1}{l}}\right)}\;],\sqrt{\log_{\vartheta }\left(1+(\vartheta -1){\left(\frac{1-\prod_{1\leq t_{1}\leq t_{2}\ldots ,\leq j_{l}\leq n}{\left(\frac{1-\overset{l}{\prod_{t=1}}\sigma_{j_{t}}}{1+(\vartheta -1)\overset{l}{\prod_{t=1}}\sigma_{j_{t}}}\right)}^{\frac{l!(n-l)!}{n!}}}{1+(\vartheta -1)\prod_{1\leq t_{1}\leq t_{2}\ldots ,\leq j_{l}\leq n}{\left(\frac{1-\overset{l}{\prod_{t=1}}\sigma_{j_{t}}}{1+(\vartheta -1)\overset{l}{\prod_{j=1}}\sigma_{j_{t}}}\right)}^{\frac{l!(n-l)!}{n!}}}\right)}^{\frac{1}{l}}\right)}\rangle ,\langle [\sqrt{1-\log_{\vartheta }\left(1+(\vartheta -1){\left(\frac{1-\prod_{1\leq t_{1}\leq t_{2}\ldots ,\leq j_{l}\leq n}{\left(\frac{1-\overset{l}{\prod_{t=1}}{\overset{˜}{\zeta }}_{j_{t}}}{1+(\vartheta -1)\overset{n}{\prod_{j=1}}{\overset{˜}{\zeta }}_{j_{t}}}\right)}^{\frac{l!(n-l)!}{n!}}}{1+(\vartheta -1)\prod_{1\leq t_{1}\leq t_{2}\ldots ,\leq j_{l}\leq n}{\left(\frac{1-\overset{l}{\prod_{t=1}}{\overset{˜}{\zeta }}_{j_{t}}}{1+(\vartheta -1)\overset{l}{\prod_{t=1}}{\overset{˜}{\zeta }}_{j_{t}}}\right)}^{\frac{l!(n-l)!}{n!}}}\right)}^{\frac{1}{l}}\right)},\sqrt{1-\log_{\vartheta }\left(1+(\vartheta -1){\left(\frac{1-\prod_{1\leq t_{1}\leq t_{2}\ldots ,\leq j_{l}\leq n}{\left(\frac{1-\overset{l}{\prod_{t=1}}{\overset{˜}{\tau }}_{j_{t}}}{1+(\vartheta -1)\overset{l}{\prod_{t=1}}{\overset{˜}{\tau }}_{j_{t}}}\right)}^{\frac{l!(n-l)!}{n!}}}{1+(\vartheta -1)\prod_{1\leq t_{1}\leq t_{2}\ldots ,\leq j_{l}\leq n}{\left(\frac{1-\overset{l}{\prod_{t=1}}{\overset{˜}{\tau }}_{j_{t}}}{1+(\vartheta -1)\overset{l}{\prod_{t=1}}{\overset{˜}{\tau }}_{j_{t}}}\right)}^{\frac{l!(n-l)!}{n!}}}\right)}^{\frac{1}{l}}\right)}\;],\sqrt{1-\log_{\vartheta }\left(1+(\vartheta -1){\left(\frac{1-\prod_{1\leq t_{1}\leq t_{2}\ldots ,\leq j_{l}\leq n}{\left(\frac{1-\overset{l}{\prod_{t=1}}\rho_{j_{t}}}{1+(\vartheta -1)\overset{l}{\prod_{t=1}}\rho_{j_{t}}}\right)}^{\frac{l!(n-l)!}{n!}}}{1+(\vartheta -1)\prod_{1\leq j_{1},\leq j_{2}\leq ,\ldots ,j_{t}\leq n}{\left(\frac{1-\overset{l}{\prod_{t=1}}\rho_{j_{t}}}{1+(\vartheta -1)\overset{l}{\prod_{t=1}}\rho_{j_{t}}}\right)}^{\frac{l!(n-l)!}{n!}}}\right)}^{\frac{1}{l}}\right)}\rangle )$$

*where*

$$\begin{array}{ccc} \zeta_{j_{t}}=\left(\frac{\vartheta^{\Lambda_{\beta_{j_{t}}}^{2}}-1}{\vartheta -1}\right),\; & \tau_{j_{t}}=\left(\frac{\vartheta^{\Gamma_{\beta_{j_{t}}}^{2}}-1}{\vartheta -1}\right),\; & \sigma_{j_{t}}=\left(\frac{\vartheta^{\xi_{\beta_{j_{t}}}^{2}}-1}{\vartheta -1}\right)\\ \; & \; & \\ {\overset{˜}{\zeta }}_{j_{t}}=\left(\frac{\vartheta^{\left(1-{\overset{˜}{\Lambda }}_{\beta_{j_{t}}}^{2}\right)}-1}{\vartheta -1}\right),\; & {\overset{˜}{\tau }}_{j_{t}}=\left(\frac{\vartheta^{\left(1-{\overset{˜}{\Gamma }}_{\beta_{j_{t}}}^{2}\right)}-1}{\vartheta -1}\right),\; & \rho_{j_{t}}=\left(\frac{\vartheta^{\left(1-\eta_{\beta_{j_{t}}}^{2}\right)}-1}{\vartheta -1}\right). \end{array}$$

Corollary 4.2 *If the vector parameter*$r=(\overset{l}{\overset{︷}{1,1,\ldots ,1}},\underset{n-l}{\underset{︸}{0,0,\ldots ,0}})$*then the* PCFFGMM *reduces to a new Maclaurin symmetric mean operator such as the Pythagorean cubic fuzzy Frank geometric MSM* (PCFFGMSM)*, which is expressed as:*

$$\mathrm{PCFFGMSM}(\beta_{1},\beta_{2},\ldots ,\beta_{n})=\frac{1}{l}\left(\underset{1\leq j_{1}\leq j_{2}\leq ,\ldots ,\leq j_{l}\leq n}{{⨂}_{F}}{\left(\overset{l}{\underset{t=1}{{⨁}_{F}}}\beta_{j_{t}}\right)}^{\frac{l!(n-l)!}{n!}}\right),$$

*where,* $\left(j_{1},j_{2},\ldots ,j_{l}\right)$ *sweep over l-tuple combinations of* {1,2,…,n}*.*

*Then,*

$$\mathrm{PCFFGMSM}(\beta_{1},\beta_{2},\ldots ,\beta_{n})=(\langle [\sqrt{1-\log_{\vartheta }\left(1+(\vartheta -1){\left(\frac{1-\prod_{1\leq t_{1}\leq t_{2}\ldots ,\leq j_{l}\leq n}{\left(\frac{1-\overset{l}{\prod_{t=1}}\zeta_{j_{t}}^{⁎}}{1+(\vartheta -1)\overset{l}{\prod_{t=1}}\zeta_{j_{t}}^{⁎}}\right)}^{\frac{l!(n-l)!}{n!}}}{1+(\vartheta -1)\prod_{1\leq t_{1}\leq t_{2}\ldots ,\leq j_{l}\leq n}{\left(\frac{1-\overset{n}{\prod_{t=1}}\zeta_{j_{t}}^{⁎}}{1+(\vartheta -1)\overset{l}{\prod_{t=1}}\zeta_{j_{t}}^{⁎}}\right)}^{\frac{l!(n-l)!}{n!}}}\right)}^{\frac{1}{l}}\right)},\sqrt{1-\log_{\vartheta }\left(1+(\vartheta -1){\left(\frac{1-\prod_{1\leq t_{1}\leq t_{2}\ldots ,\leq j_{l}\leq n}{\left(\frac{1-\overset{l}{\prod_{t=1}}\tau_{j_{t}}^{⁎}}{1+(\vartheta -1)\overset{l}{\prod_{t=1}}\tau_{j_{t}}^{⁎}}\right)}^{\frac{l!(n-l)!}{n!}}}{1+(\vartheta -1)\prod_{1\leq t_{1}\leq t_{2}\ldots ,\leq j_{l}\leq n}{\left(\frac{1-\overset{l}{\prod_{t=1}}\tau_{j_{t}}^{⁎}}{1+(\vartheta -1)\overset{l}{\prod_{t=1}}\tau_{j_{t}}^{⁎}}\right)}^{\frac{l!(n-l)!}{n!}}}\right)}^{\frac{1}{l}}\right)}\;],\sqrt{1-\log_{\vartheta }\left(1+(\vartheta -1){\left(\frac{1-\prod_{1\leq t_{1}\leq t_{2}\ldots ,\leq j_{l}\leq n}{\left(\frac{1-\overset{l}{\prod_{t=1}}\sigma_{j_{t}}^{⁎}}{1+(\vartheta -1)\overset{l}{\prod_{t=1}}\sigma_{j_{t}}^{⁎}}\right)}^{\frac{l!(n-l)!}{n!}}}{1+(\vartheta -1)\prod_{1\leq t_{1}\leq t_{2}\ldots ,\leq j_{l}\leq n}{\left(\frac{1-\overset{l}{\prod_{t=1}}\sigma_{j_{t}}^{⁎}}{1+(\vartheta -1)\overset{l}{\prod_{j=1}}\sigma_{j_{t}}^{⁎}}\right)}^{\frac{l!(n-l)!}{n!}}}\right)}^{\frac{1}{l}}\right)}\rangle ,\langle [\sqrt{\log_{\vartheta }\left(1+(\vartheta -1){\left(\frac{1-\prod_{1\leq t_{1}\leq t_{2}\ldots ,\leq j_{l}\leq n}{\left(\frac{1-\overset{l}{\prod_{t=1}}{\overset{˜}{\zeta }}_{j_{t}}^{⁎}}{1+(\vartheta -1)\overset{n}{\prod_{j=1}}{\overset{˜}{\zeta }}_{j_{t}}^{⁎}}\right)}^{\frac{l!(n-l)!}{n!}}}{1+(\vartheta -1)\prod_{1\leq t_{1}\leq t_{2}\ldots ,\leq j_{l}\leq n}{\left(\frac{1-\overset{l}{\prod_{t=1}}{\overset{˜}{\zeta }}_{j_{t}}^{⁎}}{1+(\vartheta -1)\overset{l}{\prod_{t=1}}{\overset{˜}{\zeta }}_{j_{t}}^{⁎}}\right)}^{\frac{l!(n-l)!}{n!}}}\right)}^{\frac{1}{l}}\right)},\sqrt{\log_{\vartheta }\left(1+(\vartheta -1){\left(\frac{1-\prod_{1\leq t_{1}\leq t_{2}\ldots ,\leq j_{l}\leq n}{\left(\frac{1-\overset{l}{\prod_{t=1}}{\overset{˜}{\tau }}_{j_{t}}^{⁎}}{1+(\vartheta -1)\overset{l}{\prod_{t=1}}{\overset{˜}{\tau }}_{j_{t}}^{⁎}}\right)}^{\frac{l!(n-l)!}{n!}}}{1+(\vartheta -1)\prod_{\theta (j)\in S_{n}}{\left(\frac{1-\overset{l}{\prod_{t=1}}{\overset{˜}{\tau }}_{j_{t}}^{⁎}}{1+(\vartheta -1)\overset{l}{\prod_{t=1}}{\overset{˜}{\tau }}_{j_{t}}^{⁎}}\right)}^{\frac{l!(n-l)!}{n!}}}\right)}^{\frac{1}{l}}\right)}\;],\sqrt{\log_{\vartheta }\left(1+(\vartheta -1){\left(\frac{1-\prod_{1\leq t_{1}\leq t_{2}\ldots ,\leq j_{l}\leq n}{\left(\frac{1-\overset{l}{\prod_{t=1}}\rho_{j_{t}}^{⁎}}{1+(\vartheta -1)\overset{l}{\prod_{t=1}}\rho_{j_{t}}^{⁎}}\right)}^{\frac{l!(n-l)!}{n!}}}{1+(\vartheta -1)\prod_{1\leq j_{1},\leq j_{2}\leq ,\ldots ,j_{t}\leq n}{\left(\frac{1-\overset{l}{\prod_{t=1}}\rho_{j_{t}}^{⁎}}{1+(\vartheta -1)\overset{l}{\prod_{t=1}}\rho_{j_{t}}^{⁎}}\right)}^{\frac{l!(n-l)!}{n!}}}\right)}^{\frac{1}{l}}\right)}\rangle )$$

*where*

$$\begin{array}{ccc} \zeta_{j_{t}}^{⁎}=\left(\frac{\vartheta^{\left(1-\Lambda_{\beta_{j_{t}}}^{2}\right)}-1}{\vartheta -1}\right),\; & \tau_{j_{t}}^{⁎}=\left(\frac{\vartheta^{\left(1-\Gamma_{\beta_{j_{t}}}^{2}\right)}-1}{\vartheta -1}\right),\; & \sigma_{j_{t}}^{⁎}=\left(\frac{\vartheta^{\left(1-\xi_{\beta_{j_{t}}}^{2}\right)}-1}{\vartheta -1}\right)\\ \; & \; & \\ {\overset{˜}{\zeta }}_{j_{t}}^{⁎}=\left(\frac{\vartheta^{{\overset{˜}{\Lambda }}_{\beta_{j_{t}}}^{2}}-1}{\vartheta -1}\right),\; & {\overset{˜}{\tau }}_{j_{t}}^{⁎}=\left(\frac{\vartheta^{{\overset{˜}{\Gamma }}_{\beta_{j_{t}}}^{2}}-1}{\vartheta -1}\right),\; & \rho_{j_{t}}^{⁎}=\left(\frac{\vartheta^{\eta_{\beta_{j_{t}}}^{2}}-1}{\vartheta -1}\right). \end{array}$$

Corollary 4.3 *If the vector parameter*$r=(r_{1},r_{2},0,\ldots ,0)$*then the* PCFFMM *reduces to a new Maclaurin symmetric mean operator such as the Pythagorean cubic fuzzy Frank Bonferroni mean* (PCFFBM) *operator, which is given by*

$$\mathrm{PCFFBM}(\beta_{1},\beta_{2},\ldots ,\beta_{n})={\left(\frac{1}{n(n-1)}\overset{n}{\underset{j_{1}\neq j_{2}}{\underset{j_{1},j_{2}=1}{{⨁}_{F}}}}\left(\beta_{j_{1}}^{r_{1}}{⨂}_{F}\beta_{j_{2}}^{r_{2}}\right)\right)}^{\frac{1}{r_{1}+r_{2}}},$$

*where,* $\left(j_{1},j_{2}\right)$ *sweep over* 2*-tuple combinations of* {1,2,…,n}*.*

*Then,*

$$\mathrm{PCFFBM}(\beta_{1},\beta_{2},\ldots ,\beta_{n})=(\langle [\sqrt{\log_{\vartheta }\left(1+(\vartheta -1){\left(\frac{1-\overset{n}{\underset{j_{1}\neq j_{2}}{\prod_{j_{1},j_{2}=1}}}{\left(\frac{1-\zeta_{j_{t_{1}}}^{r_{1}}\zeta_{j_{t_{2}}}^{r_{2}}}{1+(\vartheta -1)\zeta_{j_{t_{1}}}^{r_{1}}\zeta_{j_{t_{2}}}^{r_{2}}}\right)}^{\frac{1}{n(n-1)}}}{1+(\vartheta -1)\overset{n}{\underset{j_{1}\neq j_{2}}{\prod_{j_{1},j_{2}=1}}}{\left(\frac{1-\zeta_{j_{t_{1}}}^{r_{1}}\zeta_{j_{t_{2}}}^{r_{2}}}{1+(\vartheta -1)\zeta_{j_{t_{1}}}^{r_{1}}\zeta_{j_{t_{2}}}^{r_{2}}}\right)}^{\frac{1}{n(n-1)}}}\right)}^{\frac{1}{r_{1}+r_{2}}}\right)},\sqrt{\log_{\vartheta }\left(1+(\vartheta -1){\left(\frac{1-\overset{n}{\underset{j_{1}\neq j_{2}}{\prod_{j_{1},j_{2}=1}}}{\left(\frac{1-\tau_{j_{t_{1}}}^{r_{1}}\tau_{j_{t_{2}}}^{r_{2}}}{1+(\vartheta -1)\tau_{j_{t_{1}}}^{r_{1}}\tau_{j_{t_{2}}}^{r_{2}}}\right)}^{\frac{1}{n(n-1)}}}{1+(\vartheta -1)\overset{n}{\underset{j_{1}\neq j_{2}}{\prod_{j_{1},j_{2}=1}}}{\left(\frac{1-\tau_{j_{t_{1}}}^{r_{1}}\tau_{j_{t_{2}}}^{r_{2}}}{1+(\vartheta -1)\tau_{j_{t_{1}}}^{r_{1}}\tau_{j_{t_{2}}}^{r_{1}}}\right)}^{\frac{1}{n(n-1)}}}\right)}^{\frac{1}{r_{1}+r_{2}}}\right)}\;],\sqrt{\log_{\vartheta }\left(1+(\vartheta -1){\left(\frac{1-\overset{n}{\underset{j_{1}\neq j_{2}}{\prod_{j_{1},j_{2}=1}}}{\left(\frac{1-\sigma_{j_{t_{1}}}^{r_{1}}\sigma_{j_{t_{2}}}^{r_{2}}}{1+(\vartheta -1)\sigma_{j_{t_{1}}}^{r_{1}}\sigma_{j_{t_{2}}}^{r_{2}}}\right)}^{\frac{1}{n(n-1)}}}{1+(\vartheta -1)\overset{n}{\underset{j_{1}\neq j_{2}}{\prod_{j_{1},j_{2}=1}}}{\left(\frac{1-\sigma_{j_{t_{1}}}^{r_{1}}\sigma_{j_{t_{2}}}^{r_{2}}}{1+(\vartheta -1)\sigma_{j_{t_{1}}}^{r_{1}}\sigma_{j_{t_{2}}}^{r_{2}}}\right)}^{\frac{1}{n(n-1)}}}\right)}^{\frac{1}{r_{1}+r_{2}}}\right)}\rangle ,\langle [\sqrt{1-\log_{\vartheta }\left(1+(\vartheta -1){\left(\frac{1-\overset{n}{\underset{j_{1}\neq j_{2}}{\prod_{j_{1},j_{2}=1}}}{\left(\frac{1-{\overset{˜}{\zeta }}_{j_{t_{1}}}^{r_{1}}{\overset{˜}{\zeta }}_{j_{t_{2}}}^{r_{2}}}{1+(\vartheta -1){\overset{˜}{\zeta }}_{j_{t_{1}}}^{r_{1}}{\overset{˜}{\zeta }}_{j_{t_{2}}}^{r_{2}}}\right)}^{\frac{1}{n(n-1)}}}{1+(\vartheta -1)\overset{n}{\underset{j_{1}\neq j_{2}}{\prod_{j_{1},j_{2}=1}}}{\left(\frac{1-{\overset{˜}{\zeta }}_{j_{t_{1}}}^{r_{1}}{\overset{˜}{\zeta }}_{j_{t_{2}}}^{r_{2}}}{1+(\vartheta -1){\overset{˜}{\zeta }}_{j_{t_{1}}}^{r_{1}}{\overset{˜}{\zeta }}_{j_{t_{2}}}^{r_{2}}}\right)}^{\frac{1}{n(n-1)}}}\right)}^{\frac{1}{r_{1}+r_{2}}}\right)},\sqrt{1-\log_{\vartheta }\left(1+(\vartheta -1){\left(\frac{1-\overset{n}{\underset{}{\prod_{j_{1},j_{2}=1}}}{\left(\frac{1-{\overset{˜}{\tau }}_{j_{t_{1}}}^{r_{1}}{\overset{˜}{\tau }}_{j_{t_{2}}}^{r_{2}}}{1+(\vartheta -1){\overset{˜}{\tau }}_{j_{t_{1}}}^{r_{1}}{\overset{˜}{\tau }}_{j_{t_{2}}}^{r_{2}}}\right)}^{\frac{1}{n(n-1)}}}{1+(\vartheta -1)\overset{n}{\underset{j_{1}\neq j_{2}}{\prod_{j_{1},j_{2}=1}}}{\left(\frac{1-{\overset{˜}{\tau }}_{j_{t_{1}}}^{r_{1}}{\overset{˜}{\tau }}_{j_{t_{2}}}^{r_{2}}}{1+(\vartheta -1){\overset{˜}{\tau }}_{j_{t_{1}}}^{r_{1}}{\overset{˜}{\tau }}_{j_{t_{2}}}^{r_{2}}}\right)}^{\frac{1}{n(n-1)}}}\right)}^{\frac{1}{r_{1}+r_{2}}}\right)}\;],\sqrt{1-\log_{\vartheta }\left(1+(\vartheta -1){\left(\frac{1-\overset{n}{\underset{j_{1}\neq j_{2}}{\prod_{j_{1},j_{2}=1}}}{\left(\frac{1-\rho_{j_{t_{1}}}^{r_{1}}\rho_{j_{t_{2}}}^{r_{2}}}{1+(\vartheta -1)\rho_{j_{t_{1}}}^{r_{1}}\rho_{j_{t_{2}}}^{r_{2}}}\right)}^{\frac{1}{n(n-1)}}}{1+(\vartheta -1)\overset{n}{\underset{j_{1}\neq j_{2}}{\prod_{j_{1},j_{2}=1}}}{\left(\frac{1-\rho_{j_{t_{1}}}^{r_{1}}\rho_{j_{t_{2}}}^{r_{2}}}{1+(\vartheta -1)\rho_{j_{t_{1}}}^{r_{1}}\rho_{j_{t_{2}}}^{r_{2}}}\right)}^{\frac{1}{n(n-1)}}}\right)}^{\frac{1}{r_{1}+r_{2}}}\right)}\rangle )$$

*where*

$$\begin{array}{ccc} \zeta_{j_{t_{1}}}=\left(\frac{\vartheta^{\Lambda_{\beta_{j_{t_{1}}}}^{2}}-1}{\vartheta -1}\right),\; & \tau_{j_{t_{1}}}=\left(\frac{\vartheta^{\Gamma_{\beta_{j_{t_{1}}}}^{2}}-1}{\vartheta -1}\right),\; & \sigma_{j_{t_{1}}}=\left(\frac{\vartheta^{\xi_{\beta_{j_{t_{1}}}}^{2}}-1}{\vartheta -1}\right)\\ \; & \; & \\ \zeta_{j_{t_{2}}}=\left(\frac{\vartheta^{\Lambda_{\beta_{j_{t_{2}}}}^{2}}-1}{\vartheta -1}\right),\; & \tau_{j_{t_{2}}}=\left(\frac{\vartheta^{\Gamma_{\beta_{j_{t_{2}}}}^{2}}-1}{\vartheta -1}\right),\; & \sigma_{j_{t_{2}}}=\left(\frac{\vartheta^{\xi_{\beta_{j_{t_{2}}}}^{2}}-1}{\vartheta -1}\right)\\ \; & \; & \\ {\overset{˜}{\zeta }}_{j_{t_{1}}}=\left(\frac{\vartheta^{\left(1-{\overset{˜}{\Lambda }}_{\beta_{j_{t_{1}}}}^{2}\right)}-1}{\vartheta -1}\right),\; & {\overset{˜}{\tau }}_{j_{t_{1}}}=\left(\frac{\vartheta^{\left(1-{\overset{˜}{\Gamma }}_{\beta_{j_{t}}}^{2}\right)}-1}{\vartheta -1}\right),\; & \rho_{j_{t_{1}}}=\left(\frac{\vartheta^{\left(1-\eta_{\beta_{j_{t_{1}}}}^{2}\right)}-1}{\vartheta -1}\right)\\ \; & \; & \\ {\overset{˜}{\zeta }}_{j_{t_{2}}}=\left(\frac{\vartheta^{\left(1-{\overset{˜}{\Lambda }}_{\beta_{j_{t_{2}}}}^{2}\right)}-1}{\vartheta -1}\right),\; & {\overset{˜}{\tau }}_{j_{t_{2}}}=\left(\frac{\vartheta^{\left(1-{\overset{˜}{\Gamma }}_{\beta_{j_{t_{2}}}}^{2}\right)}-1}{\vartheta -1}\right),\; & \rho_{j_{t_{2}}}=\left(\frac{\vartheta^{\left(1-\eta_{\beta_{j_{t_{2}}}}^{2}\right)}-1}{\vartheta -1}\right). \end{array}$$

Corollary 4.4 *If the vector parameter*$r=(r_{1},r_{2},0,\ldots ,0)$*then* PCFFGMM *reduces to the* (PCFFGBM) *operator, which is given by*

$$\mathrm{PCFFGBM}(\beta_{1},\beta_{2},\ldots ,\beta_{n})=\frac{1}{r_{1}+r_{2}}\left(\overset{n}{\underset{j_{1}\neq j_{2}}{\underset{j_{1},j_{2}=1}{{⨂}_{F}}}}{\left(r_{1}\beta_{j_{t_{1}}}{⨁}_{F}r_{2}\beta_{j_{t_{2}}}\right)}^{\frac{1}{n(n-1)}}\right),$$

*where,* $\left(j_{1},j_{2}\right)$ *sweeps over* 2*-tuple combinations of* {1,2,…,n}*.*

*Then,*

$$\mathrm{PCFFGBM}(\beta_{1},\beta_{2},\ldots ,\beta_{n})=(\langle [\sqrt{1-\log_{\vartheta }\left(1+(\vartheta -1){\left(\frac{1-\overset{n}{\underset{j_{1}\neq j_{2}}{\prod_{j_{1},j_{2}=1}}}{\left(\frac{1-\zeta_{j_{t_{1}}}^{{⁎}^{r_{1}}}\zeta_{j_{t_{2}}}^{{⁎}^{r_{2}}}}{1+(\vartheta -1)\zeta_{j_{t_{1}}}^{{⁎}^{r_{1}}}\zeta_{j_{t_{2}}}^{{⁎}^{r_{2}}}}\right)}^{\frac{1}{n(n-1)}}}{1+(\vartheta -1)\overset{n}{\underset{j_{1}\neq j_{2}}{\prod_{j_{1},j_{2}=1}}}{\left(\frac{1-\zeta_{j_{t_{1}}}^{{⁎}^{r_{1}}}\zeta_{j_{t_{2}}}^{{⁎}^{r_{2}}}}{1+(\vartheta -1)\zeta_{j_{t_{1}}}^{{⁎}^{r_{1}}}\zeta_{j_{t_{2}}}^{{⁎}^{r_{2}}}}\right)}^{\frac{1}{n(n-1)}}}\right)}^{\frac{1}{r_{1}+r_{2}}}\right)},\sqrt{1-\log_{\vartheta }\left(1+(\vartheta -1){\left(\frac{1-\overset{n}{\underset{j_{1}\neq j_{2}}{\prod_{j_{1},j_{2}=1}}}{\left(\frac{1-\tau_{j_{t_{1}}}^{{⁎}^{r_{1}}}\tau_{j_{t_{2}}}^{{⁎}^{r_{2}}}}{1+(\vartheta -1)\tau_{j_{t_{1}}}^{r_{1}}\tau_{j_{t_{2}}}^{r_{2}}}\right)}^{\frac{1}{n(n-1)}}}{1+(\vartheta -1)\overset{n}{\underset{j_{1}\neq j_{2}}{\prod_{j_{1},j_{2}=1}}}{\left(\frac{1-\tau_{j_{t_{1}}}^{{⁎}^{r_{1}}}\tau_{j_{t_{2}}}^{{⁎}^{r_{2}}}}{1+(\vartheta -1)\tau_{j_{t_{1}}}^{{⁎}^{r_{1}}}\tau_{j_{t_{2}}}^{{⁎}^{r_{2}}}}\right)}^{\frac{1}{n(n-1)}}}\right)}^{\frac{1}{r_{1}+r_{2}}}\right)}\;],\sqrt{1-\log_{\vartheta }\left(1+(\vartheta -1){\left(\frac{1-\overset{n}{\underset{j_{1}\neq j_{2}}{\prod_{j_{1},j_{2}=1}}}{\left(\frac{1-\sigma_{j_{t_{1}}}^{{⁎}^{r_{1}}}\sigma_{j_{t_{2}}}^{{⁎}^{r_{2}}}}{1+(\vartheta -1)\sigma_{j_{t_{1}}}^{{⁎}^{r_{1}}}\sigma_{j_{t_{2}}}^{{⁎}^{r_{2}}}}\right)}^{\frac{1}{n(n-1)}}}{1+(\vartheta -1)\overset{n}{\underset{j_{1}\neq j_{2}}{\prod_{j_{1},j_{2}=1}}}{\left(\frac{1-\sigma_{j_{t_{1}}}^{{⁎}^{r_{1}}}\sigma_{j_{t_{2}}}^{{⁎}^{r_{2}}}}{1+(\vartheta -1)\sigma_{j_{t_{1}}}^{{⁎}^{r_{1}}}\sigma_{j_{t_{2}}}^{{⁎}^{r_{2}}}}\right)}^{\frac{1}{n(n-1)}}}\right)}^{\frac{1}{r_{1}+r_{2}}}\right)}\rangle ,\langle [\sqrt{\log_{\vartheta }\left(1+(\vartheta -1){\left(\frac{1-\overset{n}{\underset{j_{1}\neq j_{2}}{\prod_{j_{1},j_{2}=1}}}{\left(\frac{1-{\overset{˜}{\zeta }}_{j_{t_{1}}}^{{⁎}^{r_{1}}}{\overset{˜}{\zeta }}_{j_{t_{2}}}^{{⁎}^{r_{2}}}}{1+(\vartheta -1){\overset{˜}{\zeta }}_{j_{t_{1}}}^{{⁎}^{r_{1}}}{\overset{˜}{\zeta }}_{j_{t_{2}}}^{{⁎}^{r_{2}}}}\right)}^{\frac{1}{n(n-1)}}}{1+(\vartheta -1)\overset{n}{\underset{j_{1}\neq j_{2}}{\prod_{j_{1},j_{2}=1}}}{\left(\frac{1-{\overset{˜}{\zeta }}_{j_{t_{1}}}^{{⁎}^{r_{1}}}{\overset{˜}{\zeta }}_{j_{t_{2}}}^{{⁎}^{r_{2}}}}{1+(\vartheta -1){\overset{˜}{\zeta }}_{j_{t_{1}}}^{{⁎}^{r_{1}}}{\overset{˜}{\zeta }}_{j_{t_{2}}}^{{⁎}^{r_{2}}}}\right)}^{\frac{1}{n(n-1)}}}\right)}^{\frac{1}{r_{1}+r_{2}}}\right)},\sqrt{\log_{\vartheta }\left(1+(\vartheta -1){\left(\frac{1-\overset{n}{\underset{}{\prod_{j_{1},j_{2}=1}}}{\left(\frac{1-{\overset{˜}{\tau }}_{j_{t_{1}}}^{{⁎}^{r_{1}}}{\overset{˜}{\tau }}_{j_{t_{2}}}^{{⁎}^{r_{2}}}}{1+(\vartheta -1){\overset{˜}{\tau }}_{j_{t_{1}}}^{{⁎}^{r_{1}}}{\overset{˜}{\tau }}_{j_{t_{2}}}^{{⁎}^{r_{2}}}}\right)}^{\frac{1}{n(n-1)}}}{1+(\vartheta -1)\overset{n}{\underset{j_{1}\neq j_{2}}{\prod_{j_{1},j_{2}=1}}}{\left(\frac{1-{\overset{˜}{\tau }}_{j_{t_{1}}}^{{⁎}^{r_{1}}}{\overset{˜}{\tau }}_{j_{t_{2}}}^{{⁎}^{r_{2}}}}{1+(\vartheta -1){\overset{˜}{\tau }}_{j_{t_{1}}}^{{⁎}^{r_{1}}}{\overset{˜}{\tau }}_{j_{t_{2}}}^{{⁎}^{r_{2}}}}\right)}^{\frac{1}{n(n-1)}}}\right)}^{\frac{1}{r_{1}+r_{2}}}\right)}\;],\sqrt{\log_{\vartheta }\left(1+(\vartheta -1){\left(\frac{1-\overset{n}{\underset{j_{1}\neq j_{2}}{\prod_{j_{1},j_{2}=1}}}{\left(\frac{1-\rho_{j_{t_{1}}}^{{⁎}^{r_{1}}}\rho_{j_{t_{2}}}^{{⁎}^{r_{2}}}}{1+(\vartheta -1)\rho_{j_{t_{1}}}^{{⁎}^{r_{1}}}\rho_{j_{t_{2}}}^{{⁎}^{r_{2}}}}\right)}^{\frac{1}{n(n-1)}}}{1+(\vartheta -1)\overset{n}{\underset{j_{1}\neq j_{2}}{\prod_{j_{1},j_{2}=1}}}{\left(\frac{1-\rho_{j_{t_{1}}}^{{⁎}^{r_{1}}}\rho_{j_{t_{2}}}^{{⁎}^{r_{2}}}}{1+(\vartheta -1)\rho_{j_{t_{1}}}^{{⁎}^{r_{1}}}\rho_{j_{t_{2}}}^{{⁎}^{r_{2}}}}\right)}^{\frac{1}{n(n-1)}}}\right)}^{\frac{1}{r_{1}+r_{2}}}\right)}\rangle )$$

*where,*

$$\begin{array}{ccc} \zeta_{j_{t}}^{⁎}=\left(\frac{\vartheta^{\left(1-\Lambda_{\beta_{j_{t}}}^{2}\right)}-1}{\vartheta -1}\right),\; & \tau_{j_{t}}^{⁎}=\left(\frac{\vartheta^{\left(1-\Gamma_{\beta_{j_{t}}}^{2}\right)}-1}{\vartheta -1}\right),\; & \sigma_{j_{t}}^{⁎}=\left(\frac{\vartheta^{\left(1-\xi_{\beta_{j_{t}}}^{2}\right)}-1}{\vartheta -1}\right)\\ \; & \; & \\ {\overset{˜}{\zeta }}_{j_{t}}^{⁎}=\left(\frac{\vartheta^{{\overset{˜}{\Lambda }}_{\beta_{j_{t}}}^{2}}-1}{\vartheta -1}\right),\; & {\overset{˜}{\tau }}_{j_{t}}^{⁎}=\left(\frac{\vartheta^{{\overset{˜}{\Gamma }}_{\beta_{j_{t}}}^{2}}-1}{\vartheta -1}\right),\; & \rho_{j_{t}}^{⁎}=\left(\frac{\vartheta^{\eta_{\beta_{j_{t}}}^{2}}-1}{\vartheta -1}\right). \end{array}$$

Corollary 4.5 *If the vector parameter*r=(1,0,…,0)*then the* PCFFMM *reduces to the* (PCFFA)*, and is expressed as:*

$$\mathrm{PCFFA}(\beta_{1},\beta_{2},\ldots ,\beta_{n})=\frac{1}{n}\overset{n}{\underset{j=1}{{⨁}_{F}}}\beta_{j}$$

*Then,*

$$\mathrm{PCFFA}(\beta_{1},\beta_{2},\ldots ,\beta_{n})=(\langle [\sqrt{1-\log_{\vartheta }\left(1+(\vartheta -1)\overset{n}{\prod_{j=1}}{\left(\frac{1-\zeta_{j}}{1+(\vartheta -1)\zeta_{j}}\right)}^{\frac{1}{n}}\right)},\sqrt{1-\log_{\vartheta }\left(1+(\vartheta -1)\overset{n}{\prod_{j=1}}{\left(\frac{1-\tau_{j}}{1+(\vartheta -1)\tau_{j}}\right)}^{\frac{1}{n}}\right)}\;],\sqrt{1-\log_{\vartheta }\left(1+(\vartheta -1)\overset{n}{\prod_{j=1}}{\left(\frac{1-\sigma_{j}}{1+(\vartheta -1)\sigma_{j}}\right)}^{\frac{1}{n}}\right)}\rangle ,\langle [\sqrt{\log_{\vartheta }\left(1+(\vartheta -1)\overset{n}{\prod_{j=1}}{\left(\frac{1-{\overset{˜}{\zeta }}_{j}}{1+(\vartheta -1){\overset{˜}{\zeta }}_{j}}\right)}^{\frac{1}{n}}\right)},\sqrt{\log_{\vartheta }\left(1+(\vartheta -1)\overset{n}{\prod_{j=1}}{\left(\frac{1-{\overset{˜}{\tau }}_{j}}{1+(\vartheta -1){\overset{˜}{\tau }}_{j}}\right)}^{\frac{1}{n}}\right)}\;],\sqrt{\log_{\vartheta }\left(1+(\vartheta -1)\overset{n}{\prod_{j=1}}{\left(\frac{1-\rho_{j}}{1+(\vartheta -1)\rho_{j}}\right)}^{\frac{1}{n}}\right)}\rangle )$$

*where,*

$$\begin{array}{ccc} \zeta_{j}=\left(\frac{\vartheta^{\Lambda_{\beta_{j}}^{2}}-1}{\vartheta -1}\right),\; & \tau_{j_{t}}=\left(\frac{\vartheta^{\Gamma_{\beta_{j}}^{2}}-1}{\vartheta -1}\right),\; & \sigma_{j}=\left(\frac{\vartheta^{\xi_{\beta_{j}}^{2}}-1}{\vartheta -1}\right)\\ \; & \; & \\ {\overset{˜}{\zeta }}_{j}=\left(\frac{\vartheta^{\left(1-{\overset{˜}{\Lambda }}_{\beta_{j}}^{2}\right)}-1}{\vartheta -1}\right),\; & {\overset{˜}{\tau }}_{j}=\left(\frac{\vartheta^{\left(1-{\overset{˜}{\Gamma }}_{\beta_{j}}^{2}\right)}-1}{\vartheta -1}\right),\; & \rho_{j}=\left(\frac{\vartheta^{\left(1-\eta_{\beta_{j}}^{2}\right)}-1}{\vartheta -1}\right). \end{array}$$

Corollary 4.6 *If the vector parameter*r=(1,0,…,0)*then the* PCFFGMM *reduces to the* (PCFFG)*, which is given by*

$$\mathrm{PCFFG}(\beta_{1},\beta_{2},\ldots ,\beta_{n})=\overset{n}{\underset{j=1}{{⨂}_{F}}}\beta_{j}^{\frac{1}{n}}$$

*Then,*

$$\mathrm{PCFFG}(\beta_{1},\beta_{2},\ldots ,\beta_{n})=(\langle [\sqrt{\log_{\vartheta }\left(1+(\vartheta -1)\overset{n}{\prod_{j=1}}{\left(\frac{1-\zeta_{j}^{⁎}}{1+(\vartheta -1)\zeta_{j}^{⁎}}\right)}^{\frac{1}{n}}\right)},\sqrt{\log_{\vartheta }\left(1+(\vartheta -1)\overset{n}{\prod_{j=1}}{\left(\frac{1-\tau_{j}^{⁎}}{1+(\vartheta -1)\tau_{j}^{⁎}}\right)}^{\frac{1}{n}}\right)}\;],\sqrt{\log_{\vartheta }\left(1+(\vartheta -1)\overset{n}{\prod_{j=1}}{\left(\frac{1-\sigma_{j}^{⁎}}{1+(\vartheta -1)\sigma_{j}^{⁎}}\right)}^{\frac{1}{n}}\right)}\rangle ,\langle [\sqrt{1-\log_{\vartheta }\left(1+(\vartheta -1)\overset{n}{\prod_{j=1}}{\left(\frac{1-{\overset{˜}{\zeta }}_{j}^{⁎}}{1+(\vartheta -1){\overset{˜}{\zeta }}_{j}^{⁎}}\right)}^{\frac{1}{n}}\right)},\sqrt{1-\log_{\vartheta }\left(1+(\vartheta -1)\overset{n}{\prod_{j=1}}{\left(\frac{1-{\overset{˜}{\tau }}_{j}^{⁎}}{1+(\vartheta -1){\overset{˜}{\tau }}_{j}^{⁎}}\right)}^{\frac{1}{n}}\right)}\;],\sqrt{1-\log_{\vartheta }\left(1+(\vartheta -1)\overset{n}{\prod_{j=1}}{\left(\frac{1-{\overset{˜}{\rho }}_{j}^{⁎}}{1+(\vartheta -1){\overset{˜}{\rho }}_{j}^{⁎}}\right)}^{\frac{1}{n}}\right)}\rangle )$$

*where,*

$$\begin{array}{ccc} \zeta_{j}^{⁎}=\left(\frac{\vartheta^{\left(1-\Lambda_{\beta_{j}}^{2}\right)}-1}{\vartheta -1}\right),\; & \tau_{j}^{⁎}=\left(\frac{\vartheta^{\left(1-\Gamma_{\beta_{j}}^{2}\right)}-1}{\vartheta -1}\right),\; & \sigma_{j}^{⁎}=\left(\frac{\vartheta^{\left(1-\xi_{\beta_{j}}^{2}\right)}-1}{\vartheta -1}\right)\\ \; & \; & \\ {\overset{˜}{\zeta }}_{j}^{⁎}=\left(\frac{\vartheta^{{\overset{˜}{\Lambda }}_{\beta_{j}}^{2}}-1}{\vartheta -1}\right),\; & {\overset{˜}{\tau }}_{j}^{⁎}=\left(\frac{\vartheta^{{\overset{˜}{\Gamma }}_{\beta_{j}}^{2}}-1}{\vartheta -1}\right),\; & \rho_{j}^{⁎}=\left(\frac{\vartheta^{\eta_{\beta_{j}}^{2}}-1}{\vartheta -1}\right). \end{array}$$

Remark 1 From Corollary 4.1, Corollary 4.2, it is seen that the PCFFMM, and the PCFFGMM operators can be reduced to some novel Frank MSM operators, e.g. the Pythagorean cubic fuzzy Frank MSM (PCFFMSM) and Pythagorean cubic fuzzy Frank geometric MSM (PCFFGMSM) operators.

Remark 2 From Corollary 4.3, Corollary 4.4, it is seen that the PCFFMM, and PCFFGMM operators can be restricted to some novel Frank Bonferroni mean operators, namely, Pythagorean cubic fuzzy Frank BM (PCFFBM), and Pythagorean cubic fuzzy Frank geometric BM (PCFFGBM) operators.

Remark 3 Further, Corollary 4.5, Corollary 4.6, indicate that the PCFFMM, and PCFFGMM operators can be reduced to the novel Pythagorean cubic fuzzy Frank average (PCFFA), and the Pythagorean cubic fuzzy Frank geometric (PCFFG) operators.

### 4.1 Properties of PCFFMM and PCFFGMM operators

Theorem 4.5 **Idempotency**

*Let* $\beta_{j}=\left\{\left(\langle [\Lambda_{\beta_{j}},\Gamma_{\beta_{j}}],\xi_{\beta_{j}}\rangle ,\langle [{\overset{˜}{\Lambda }}_{\beta_{j}},{\overset{˜}{\Gamma }}_{\beta_{j}}],\eta_{\beta_{j}}\rangle \right)|1⩽j⩽n\right\}$ *be a set of PCFNs on* V *. If* $\beta_{j}=\beta \;\forall j(1⩽j⩽n)$ *, then*

PCFFMM(β,β,…,β)=PCFFGMM(β,β,…,β)=β

Theorem 4.6 **Commutativity**

*Let* $\beta_{j=}\left(\langle [\Lambda_{\beta_{j}},\Gamma_{\beta_{j}}],\xi_{\beta_{j}}\rangle ,\langle [{\overset{˜}{\Lambda }}_{\beta_{j}},{\overset{˜}{\Gamma }}_{\beta_{j}}],\eta_{\beta_{j}}\rangle \right)$ (1⩽j⩽n) *be a set of PCFNs on* V *, and* $\beta_{j}^{\prime }=\left(\langle [\Lambda_{\beta_{j}^{\prime }},\Gamma_{\beta_{j}^{\prime }}],\xi_{\beta_{j}^{\prime }}\rangle ,\langle [{\overset{˜}{\Lambda }}_{\beta_{j}^{\prime }},{\overset{˜}{\Gamma }}_{\beta_{j}^{\prime }}],\eta_{\beta_{j}^{\prime }}\rangle \right)$ (1⩽j⩽n) *be a permutation of* $\beta_{j}$ *. Then*

$$\mathrm{PCFFMM}(\beta_{1},\beta_{2},\ldots ,\beta_{n})=\mathrm{PCFFMM}(\beta_{1}^{\prime },\beta_{2}^{\prime },\ldots ,\beta_{n}^{\prime })\mathrm{PCFFGMM}(\beta_{1},\beta_{2},\ldots ,\beta_{n})=\mathrm{PCFFGMM}(\beta_{1}^{\prime },\beta_{2}^{\prime },\ldots ,\beta_{n}^{\prime })$$

Theorem 4.7 **Comonotonicity**

*If*$\beta_{j}=\left(\langle [\Lambda_{\beta_{j}},\Gamma_{\beta_{j}}],\xi_{\beta_{j}}\rangle ,\langle [{\overset{˜}{\Lambda }}_{\beta_{j}},{\overset{˜}{\Gamma }}_{\beta_{j}}],\eta_{\beta_{j}}\rangle \right)$(1⩽j⩽n)*and*$\zeta_{j}=\left(\langle [\Lambda_{\zeta_{j}},\Gamma_{\zeta_{j}}],\xi_{\zeta_{j}}\rangle ,\langle [{\overset{˜}{\Lambda }}_{\zeta_{j}},{\overset{˜}{\Gamma }}_{\zeta_{j}}],\eta_{\zeta_{j}}\rangle \right)\;(1⩽j⩽n)$*be two sets of* PCFNs *on* V*, such that* $\Lambda_{\beta_{j}}⩽\Lambda_{\zeta_{j}},\Gamma_{\beta_{j}}⩾\Gamma_{\zeta_{j}},\xi_{\beta_{j}}⩾\xi_{\zeta_{j}}$ *and* ${\overset{˜}{\Lambda }}_{\beta_{j}}⩽{\overset{˜}{\Lambda }}_{\zeta_{j}},{\overset{˜}{\Gamma }}_{\beta_{j}}⩾{\overset{˜}{\Gamma }}_{\zeta_{j}},\eta_{\beta_{j}}⩾\eta_{\zeta_{j}}$ *for all j, then*

$$\mathrm{PCFFMM}(\beta_{1},\beta_{2},\ldots ,\beta_{n})≼\mathrm{PCFFMM}(\zeta_{1},\zeta_{2},\ldots ,\zeta_{n})\mathrm{PCFFGMM}(\beta_{1},\beta_{2},\ldots ,\beta_{n})≼\mathrm{PCFFGMM}(\zeta_{1},\zeta_{2},\ldots ,\zeta_{n})$$

Theorem 4.8 **Bounded**

*Let*$\beta_{j}=\left(\langle [\Lambda_{\beta_{j}},\Gamma_{\beta_{j}}],\xi_{\beta_{j}}\rangle ,\langle [{\overset{˜}{\Lambda }}_{\beta_{j}},{\overset{˜}{\Gamma }}_{\beta_{j}}],\eta_{\beta_{j}}\rangle \right)$(1⩽j⩽n)*be a set of* PCFNs *on* V*. Then,*

$$\beta^{-}≼\mathrm{PCFFMM}(\beta_{1},\beta_{2},\ldots ,\beta_{n})≼\beta^{+}\;\beta^{-}≼\mathrm{PCFFGMM}(\beta_{1},\beta_{2},\ldots ,\beta_{n})≼\beta^{+}$$

*where,*

$$\begin{array}{c} \beta^{-}=\left(\langle [\underset{j}{\min}(\Lambda_{\beta_{j}}),\underset{j}{\max}(\Gamma_{\beta_{j}})],\underset{j}{\max}(\xi_{\beta_{j}})\rangle ,\langle [\underset{j}{\min}({\overset{˜}{\Lambda }}_{\beta_{j}}),\underset{j}{\max}({\overset{˜}{\Gamma }}_{\beta_{j}})],\underset{j}{\max}(\eta_{\beta_{j}})\rangle \right)\\ \beta^{+}=\left(\langle [\underset{j}{\max}(\Lambda_{\beta_{j}}),\underset{j}{\min}(\Gamma_{\beta_{j}})],\underset{j}{\min}(\xi_{\beta_{j}})\rangle ,\langle [\underset{j}{\max}({\overset{˜}{\Lambda }}_{\beta_{j}}),\underset{j}{\min}({\overset{˜}{\Gamma }}_{\beta_{j}})],\underset{j}{\min}(\eta_{\beta_{j}})\rangle \right). \end{array}$$

## 5 An MCDM method based on PCFFMM and PCFFGMM

This section introduces an MCDM method using the PCFFMM and PCFFGMM operators.

Suppose that $V=\{v_{1},v_{2},\ldots ,v_{m}\}$ be a collection of choices and $C=\{C_{1},C_{2},\ldots ,C_{n}\}$ be a group of criteria and a set $D=\{D_{1},D_{2},\ldots ,D_{l}\}$ of *l* decision makers evaluates the choices $v_{i}\;(i=1,2,\ldots ,m)$. Assuming that evaluation information of each choice $v_{i}$ relative to each criterion $C_{j}$ is expressed as a PCFN.

The decision making procedure utilizing PCFFMM or PCFFGMM is outlined in the following:

***Step 1.*** Express assessment information of choices $v_{i}\;(1⩽i⩽m)$ corresponding to $C_{j}\;(1⩽j⩽n)$ into PCFN fuzzy decision matrices $E^{\left(s\right)}={\left[\beta_{ij}^{\left(s\right)}\right]}_{m\times n}$, where each $\beta_{ij}^{\left(s\right)}\;(1⩽i⩽m;1⩽j⩽n;1⩽s⩽l)$ is a PCFN, and is given by
  $$\beta_{ij}^{\left(s\right)}=\left(\langle A_{\beta_{ij}^{\left(s\right)}},\xi_{\beta_{ij}^{\left(s\right)}}\rangle ,\langle {\overset{˜}{A}}_{\beta_{ij}^{\left(s\right)}},\eta_{\beta_{ij}^{\left(s\right)}}\rangle \right)$$
  where, $A_{\beta_{ij}^{\left(s\right)}}=[\Lambda_{\beta_{ij}^{\left(s\right)}},\Gamma_{\beta_{ij}^{\left(s\right)}}],{\overset{˜}{A}}_{\beta_{ij}^{\left(s\right)}}=[{\overset{˜}{\Lambda }}_{\beta_{ij}^{\left(s\right)}},{\overset{˜}{\Gamma }}_{\beta_{ij}^{\left(s\right)}}]$ such that
  $$0⩽{\left(\sup(A_{C}(v_{i}))\right)}^{2}+{\left(\sup({\overset{˜}{A}}_{C}(v_{i}))\right)}^{2}⩽1,0⩽{\left(\xi_{C}(v_{i})\right)}^{2}+{\left(\eta_{C}(v_{i})\right)}^{2}⩽1.$$
***Step 2.*** Determine the normalized PCFN decision matrices $R^{\left(s\right)}={\left[\alpha_{ij}^{\left(s\right)}\right]}_{m\times n}$ by converting the decision matrices $E^{\left(s\right)}\;(1⩽s⩽l)$ for each DM $d_{s}$ using Equation set (3) depending on cost-type [74] and benefit-type [74] criteria.
  (3) $$\alpha_{ij}^{\left(s\right)}=\left\{ \begin{array}{cc} \beta_{ij}^{\left(s\right)},\; & \text{for benefit-type [74] criteria}\;C_{j}\\ \; & \\ {\left(\beta_{ij}^{\left(s\right)}\right)}^{c},\; & \text{for cost-type [74] criteria}\;C_{j} \end{array}\right.$$
  here ${\left(\beta_{ij}^{\left(s\right)}\right)}^{c}=\left(\langle {\overset{˜}{A}}_{\beta_{ij}^{\left(s\right)}},\eta_{\beta_{ij}^{\left(s\right)}}\rangle ,\langle A_{\beta_{ij}^{\left(s\right)}},\xi_{\beta_{ij}^{\left(s\right)}}\rangle \right)$.
***Step 3.*** Utilize Equation (1) (or Equation (2)) of the PCFFMM (or PCFFGMM) operator to aggregate each normalized PCFN decision matrices $R^{\left(s\right)}={\left[\alpha_{ij}^{\left(s\right)}\right]}_{m\times n}$ into the collective normalized PCFN decision matrix $R={\left[\alpha_{ij}\right]}_{m\times n}$.
***Step 4.*** Utilizing the PCFFMM (or PCFFGMM) operator aggregate all the preference values $\alpha_{ij}$ of the collective normalized PCFN decision matrix $R={\left[\alpha_{ij}\right]}_{m\times n}$ into the aggregated assessment values $\alpha_{i}\;(1⩽i⩽m)$ corresponding to each choice $v_{i}\;(1⩽i⩽m)$.
***Step 5.*** Determine the score $S^{i}=S(\alpha_{i})$ and accuracy $H^{i}=H(\alpha_{i})$ using Definition 2.7 of the aggregated values $\alpha_{i}$ for re-arranging the choices $v_{i}\;(1⩽i⩽m)$ and choose the optimal.
***Step 6.*** End.

## 6 Demonstrative example

Suppose a investment firm intends to select a suitable business partner from a list of four available choices $v_{i}\;(i=1,2,3,4)$ under the criteria,

(i) $C_{1}$: Investment strength,
(ii) $C_{2}$: Quality assurance,
(iii) $C_{3}$: Time management,
(iv) $C_{4}$: Trading potential.

Also, suppose that a group of three DMs $\left\{D_{1},D_{2},D_{3}\right\}$ evaluates the four choices $\left\{v_{1},v_{2},v_{3},v_{4}\right\}$ based on four criteria $\left\{C_{1},C_{2},C_{3},C_{4}\right\}$.

***Step 1.*** The assessment data of the four choices $v_{i}\;(i=1,2,3,4)$ relative to each criterion $C_{j}\;(j=1,2,3,4)$ are described by the three PCFN matrices $E^{\left(s\right)}={\left[\beta_{ij}^{\left(s\right)}\right]}_{4\times 4}\;(s=1,2,3)$ are expressed in Table 2, Table 3, Table 4.

**Table 2:** The PCFN decision matrix *E*^(1)^ due to DM $D_{1}$.

	$C_{1}$	$C_{2}$	$C_{3}$	$C_{4}$
$v_{1}$	$\left(\begin{array}{c} \langle [0.7,0.8],0.6\rangle ,\\ \langle [0.5,0.6],0.7\rangle \end{array}\right)$	$\left(\begin{array}{c} \langle [0.6,0.7],0.5\rangle ,\\ \langle [0.5,0.7],0.6\rangle \end{array}\right)$	$\left(\begin{array}{c} \langle [0.6,0.8],0.6\rangle ,\\ \langle [0.5,0.7],0.5\rangle \end{array}\right)$	$\left(\begin{array}{c} \langle [0.4,0.5],0.6\rangle ,\\ \langle [0.7,0.9],0.8\rangle \end{array}\right)$
$v_{2}$	$\left(\begin{array}{c} \langle [0.5,0.6],0.5\rangle ,\\ \langle [0.4,0.6],0.6\rangle \end{array}\right)$	$\left(\begin{array}{c} \langle [0.4,0.5],0.6\rangle ,\\ \langle [0.8,0.9],0.7\rangle \end{array}\right)$	$\left(\begin{array}{c} \langle [0.6,0.7],0.8\rangle ,\\ \langle [0.5,0.6],0.7\rangle \end{array}\right)$	$\left(\begin{array}{c} \langle [0.6,0.7],0.6\rangle ,\\ \langle [0.4,0.6],0.7\rangle \end{array}\right)$
$v_{3}$	$\left(\begin{array}{c} \langle [0.5,0.6],0.7\rangle ,\\ \langle [0.4,0.6],0.5\rangle \end{array}\right)$	$\left(\begin{array}{c} \langle [0.5,0.7],0.6\rangle ,\\ \langle [0.5,0.6],0.7\rangle \end{array}\right)$	$\left(\begin{array}{c} \langle [0.6,0.8],0.4\rangle ,\\ \langle [0.5,0.6],0.8\rangle \end{array}\right)$	$\left(\begin{array}{c} \langle [0.6,0.7],0.8\rangle ,\\ \langle [0.5,0.6],0.7\rangle \end{array}\right)$
$v_{4}$	$\left(\begin{array}{c} \langle [0.6,0.8],0.7\rangle ,\\ \langle [0.4,0.6],0.7\rangle \end{array}\right)$	$\left(\begin{array}{c} \langle [0.6,0.7],0.8\rangle ,\\ \langle [0.5,0.6],0.5\rangle \end{array}\right)$	$\left(\begin{array}{c} \langle [0.5,0.7],0.8\rangle ,\\ \langle [0.4,0.6],0.6\rangle \end{array}\right)$	$\left(\begin{array}{c} \langle [0.4,0.5],0.7\rangle ,\\ \langle [0.7,0.8],0.9\rangle \end{array}\right)$

**Table 3:** The PCFN decision matrix *E*^(2)^ due to DM $D_{2}$.

	$C_{1}$	$C_{2}$	$C_{3}$	$C_{4}$
$v_{1}$	$\left(\begin{array}{c} \langle [0.4,0.5],0.6\rangle ,\\ \langle [0.7,0.8],0.9\rangle \end{array}\right)$	$\left(\begin{array}{c} \langle [0.3,0.4],0.5\rangle ,\\ \langle [0.6,0.8],0.7\rangle \end{array}\right)$	$\left(\begin{array}{c} \langle [0.8,0.9],0.7\rangle ,\\ \langle [0.3,0.4],0.6\rangle \end{array}\right)$	$\left(\begin{array}{c} \langle [0.7,0.8],0.7\rangle ,\\ \langle [0.4,0.5],0.6\rangle \end{array}\right)$
$v_{2}$	$\left(\begin{array}{c} \langle [0.5,0.6],0.7\rangle ,\\ \langle [0.6,0.7],0.5\rangle \end{array}\right)$	$\left(\begin{array}{c} \langle [0.3,0.5],0.7\rangle ,\\ \langle [0.8,0.9],0.6\rangle \end{array}\right)$	$\left(\begin{array}{c} \langle [0.6,0.8],0.5\rangle ,\\ \langle [0.4,0.5],0.8\rangle \end{array}\right)$	$\left(\begin{array}{c} \langle [0.7,0.8],0.8\rangle ,\\ \langle [0.3,0.5],0.7\rangle \end{array}\right)$
$v_{3}$	$\left(\begin{array}{c} \langle [0.7,0.8],0.5\rangle ,\\ \langle [0.4,0.5],0.6\rangle \end{array}\right)$	$\left(\begin{array}{c} \langle [0.4,0.5],0.6\rangle ,\\ \langle [0.7,0.8],0.6\rangle \end{array}\right)$	$\left(\begin{array}{c} \langle [0.5,0.8],0.7\rangle ,\\ \langle [0.6,0.8],0.6\rangle \end{array}\right)$	$\left(\begin{array}{c} \langle [0.5,0.6],0.5\rangle ,\\ \langle [0.5,0.7],0.6\rangle \end{array}\right)$
$v_{4}$	$\left(\begin{array}{c} \langle [0.5,0.8],0.7\rangle ,\\ \langle [0.4,0.6],0.5\rangle \end{array}\right)$	$\left(\begin{array}{c} \langle [0.7,0.8],0.4\rangle ,\\ \langle [0.4,0.5],0.7\rangle \end{array}\right)$	$\left(\begin{array}{c} \langle [0.6,0.8],0.8\rangle ,\\ \langle [0.5,0.7],0.3\rangle \end{array}\right)$	$\left(\begin{array}{c} \langle [0.3,0.5],0.6\rangle ,\\ \langle [0.7,0.8],0.6\rangle \end{array}\right)$

**Table 4:** The PCFN decision matrix *E*^(3)^ due to DM $D_{3}$.

	$C_{1}$	$C_{2}$	$C_{3}$	$C_{4}$
$v_{1}$	$\left(\begin{array}{c} \langle [0.5,0.7],0.6\rangle ,\\ \langle [0.5,0.6],0.5\rangle \end{array}\right)$	$\left(\begin{array}{c} \langle [0.6,0.8],0.7\rangle ,\\ \langle [0.5,0.6],0.8\rangle \end{array}\right)$	$\left(\begin{array}{c} \langle [0.3,0.5],0.5\rangle ,\\ \langle [0.5,0.6],0.7\rangle \end{array}\right)$	$\left(\begin{array}{c} \langle [0.5,0.7],0.8\rangle ,\\ \langle [0.6,0.7],0.4\rangle \end{array}\right)$
$v_{2}$	$\left(\begin{array}{c} \langle [0.6,0.8],0.4\rangle ,\\ \langle [0.4,0.5],0.6\rangle \end{array}\right)$	$\left(\begin{array}{c} \langle [0.4,0.5],0.6\rangle ,\\ \langle [0.5,0.8],0.6\rangle \end{array}\right)$	$\left(\begin{array}{c} \langle [0.4,0.7],0.5\rangle ,\\ \langle [0.5,0.6],0.4\rangle \end{array}\right)$	$\left(\begin{array}{c} \langle [0.6,0.7],0.5\rangle ,\\ \langle [0.3,0.6],0.5\rangle \end{array}\right)$
$v_{3}$	$\left(\begin{array}{c} \langle [0.5,0.8],0.7\rangle ,\\ \langle [0.3,0.5],0.4\rangle \end{array}\right)$	$\left(\begin{array}{c} \langle [0.5,0.6],0.5\rangle ,\\ \langle [0.3,0.6],0.5\rangle \end{array}\right)$	$\left(\begin{array}{c} \langle [0.6,0.7],0.5\rangle ,\\ \langle [0.5,0.6],0.3\rangle \end{array}\right)$	$\left(\begin{array}{c} \langle [0.6,0.8],0.6\rangle ,\\ \langle [0.4,0.5],0.4\rangle \end{array}\right)$
$v_{4}$	$\left(\begin{array}{c} \langle [0.6,0.7],0.6\rangle ,\\ \langle [0.5,0.7],0.5\rangle \end{array}\right)$	$\left(\begin{array}{c} \langle [0.7,0.8],0.4\rangle ,\\ \langle [0.3,0.5],0.6\rangle \end{array}\right)$	$\left(\begin{array}{c} \langle [0.7,0.8],0.6\rangle ,\\ \langle [0.4,0.5],0.6\rangle \end{array}\right)$	$\left(\begin{array}{c} \langle [0.6,0.8],0.9\rangle ,\\ \langle [0.4,0.6],0.3\rangle \end{array}\right)$
***Step 2.*** Clearly, $C_{2},C_{3}$ are the benefit type [74] criteria, and $C_{1}$ the cost type [74] criteria. Utilizing Equation set (3) the normalized PCFN decision matrices $N^{\left(s\right)}$, (s=1,2,3) are obtained as shown in Table 5, Table 6, Table 7.

**Table 5:** The normalized PCFN decision matrix *N*^(1)^ due to DM $D_{1}$.

	$C_{1}$	$C_{2}$	$C_{3}$	$C_{4}$
$v_{1}$	$\left(\begin{array}{c} \langle [0.5,0.6],0.7\rangle ,\\ \langle [0.7,0.8],0.6\rangle \end{array}\right)$	$\left(\begin{array}{c} \langle [0.6,0.7],0.5\rangle ,\\ \langle [0.5,0.7],0.6\rangle \end{array}\right)$	$\left(\begin{array}{c} \langle [0.6,0.8],0.6\rangle ,\\ \langle [0.5,0.7],0.5\rangle \end{array}\right)$	$\left(\begin{array}{c} \langle [0.4,0.5],0.6\rangle ,\\ \langle [0.7,0.9],0.8\rangle \end{array}\right)$
$v_{2}$	$\left(\begin{array}{c} \langle [0.4,0.6],0.6\rangle ,\\ \langle [0.5,0.6],0.5\rangle \end{array}\right)$	$\left(\begin{array}{c} \langle [0.4,0.5],0.6\rangle ,\\ \langle [0.8,0.9],0.7\rangle \end{array}\right)$	$\left(\begin{array}{c} \langle [0.6,0.7],0.8\rangle ,\\ \langle [0.5,0.6],0.7\rangle \end{array}\right)$	$\left(\begin{array}{c} \langle [0.6,0.7],0.6\rangle ,\\ \langle [0.4,0.6],0.7\rangle \end{array}\right)$
$v_{3}$	$\left(\begin{array}{c} \langle [0.4,0.6],0.5\rangle ,\\ \langle [0.5,0.6],0.7\rangle \end{array}\right)$	$\left(\begin{array}{c} \langle [0.5,0.7],0.6\rangle ,\\ \langle [0.5,0.6],0.7\rangle \end{array}\right)$	$\left(\begin{array}{c} \langle [0.6,0.8],0.4\rangle ,\\ \langle [0.5,0.6],0.8\rangle \end{array}\right)$	$\left(\begin{array}{c} \langle [0.6,0.7],0.8\rangle ,\\ \langle [0.5,0.6],0.7\rangle \end{array}\right)$
$v_{4}$	$\left(\begin{array}{c} \langle [0.4,0.6],0.7\rangle ,\\ \langle [0.6,0.8],0.7\rangle \end{array}\right)$	$\left(\begin{array}{c} \langle [0.6,0.7],0.8\rangle ,\\ \langle [0.5,0.6],0.5\rangle \end{array}\right)$	$\left(\begin{array}{c} \langle [0.5,0.7],0.8\rangle ,\\ \langle [0.4,0.6],0.6\rangle \end{array}\right)$	$\left(\begin{array}{c} \langle [0.4,0.5],0.7\rangle ,\\ \langle [0.7,0.8],0.9\rangle \end{array}\right)$

**Table 6:** The normalized PCFN decision matrix *N*^(2)^ due to DM $D_{2}$.

	$C_{1}$	$C_{2}$	$C_{3}$	$C_{4}$
$v_{1}$	$\left(\begin{array}{c} \langle [0.7,0.8],0.9\rangle ,\\ \langle [0.4,0.5],0.6\rangle \end{array}\right)$	$\left(\begin{array}{c} \langle [0.3,0.4],0.5\rangle ,\\ \langle [0.6,0.8],0.7\rangle \end{array}\right)$	$\left(\begin{array}{c} \langle [0.8,0.9],0.7\rangle ,\\ \langle [0.3,0.4],0.6\rangle \end{array}\right)$	$\left(\begin{array}{c} \langle [0.7,0.8],0.7\rangle ,\\ \langle [0.4,0.5],0.6\rangle \end{array}\right)$
$v_{2}$	$\left(\begin{array}{c} \langle [0.6,0.7],0.5\rangle ,\\ \langle [0.5,0.6],0.7\rangle \end{array}\right)$	$\left(\begin{array}{c} \langle [0.3,0.5],0.7\rangle ,\\ \langle [0.8,0.9],0.6\rangle \end{array}\right)$	$\left(\begin{array}{c} \langle [0.6,0.8],0.5\rangle ,\\ \langle [0.4,0.5],0.8\rangle \end{array}\right)$	$\left(\begin{array}{c} \langle [0.7,0.8],0.8\rangle ,\\ \langle [0.3,0.5],0.7\rangle \end{array}\right)$
$v_{3}$	$\left(\begin{array}{c} \langle [0.4,0.5],0.6\rangle ,\\ \langle [0.7,0.8],0.5\rangle \end{array}\right)$	$\left(\begin{array}{c} \langle [0.4,0.5],0.6\rangle ,\\ \langle [0.7,0.8],0.6\rangle \end{array}\right)$	$\left(\begin{array}{c} \langle [0.5,0.8],0.7\rangle ,\\ \langle [0.6,0.8],0.6\rangle \end{array}\right)$	$\left(\begin{array}{c} \langle [0.5,0.6],0.5\rangle ,\\ \langle [0.5,0.7],0.6\rangle \end{array}\right)$
$v_{4}$	$\left(\begin{array}{c} \langle [0.4,0.6],0.5\rangle ,\\ \langle [0.5,0.8],0.7\rangle \end{array}\right)$	$\left(\begin{array}{c} \langle [0.7,0.8],0.4\rangle ,\\ \langle [0.4,0.5],0.7\rangle \end{array}\right)$	$\left(\begin{array}{c} \langle [0.6,0.8],0.8\rangle ,\\ \langle [0.5,0.7],0.3\rangle \end{array}\right)$	$\left(\begin{array}{c} \langle [0.3,0.5],0.6\rangle ,\\ \langle [0.7,0.8],0.6\rangle \end{array}\right)$

**Table 7:** The normalized PCFN decision matrix *N*^(3)^ due to DM $D_{3}$.

	$C_{1}$	$C_{2}$	$C_{3}$	$C_{4}$
$v_{1}$	$\left(\begin{array}{c} \langle [0.5,0.6],0.5\rangle ,\\ \langle [0.5,0.7],0.6\rangle \end{array}\right)$	$\left(\begin{array}{c} \langle [0.6,0.8],0.7\rangle ,\\ \langle [0.5,0.6],0.8\rangle \end{array}\right)$	$\left(\begin{array}{c} \langle [0.3,0.5],0.5\rangle ,\\ \langle [0.5,0.6],0.7\rangle \end{array}\right)$	$\left(\begin{array}{c} \langle [0.5,0.7],0.8\rangle ,\\ \langle [0.6,0.7],0.4\rangle \end{array}\right)$
$v_{2}$	$\left(\begin{array}{c} \langle [0.4,0.5],0.6\rangle ,\\ \langle [0.6,0.8],0.4\rangle \end{array}\right)$	$\left(\begin{array}{c} \langle [0.4,0.5],0.6\rangle ,\\ \langle [0.5,0.8],0.6\rangle \end{array}\right)$	$\left(\begin{array}{c} \langle [0.4,0.7],0.5\rangle ,\\ \langle [0.5,0.6],0.4\rangle \end{array}\right)$	$\left(\begin{array}{c} \langle [0.6,0.7],0.5\rangle ,\\ \langle [0.3,0.6],0.5\rangle \end{array}\right)$
$v_{3}$	$\left(\begin{array}{c} \langle [0.3,0.5],0.4\rangle ,\\ \langle [0.5,0.8],0.7\rangle \end{array}\right)$	$\left(\begin{array}{c} \langle [0.5,0.6],0.5\rangle ,\\ \langle [0.3,0.6],0.5\rangle \end{array}\right)$	$\left(\begin{array}{c} \langle [0.6,0.7],0.5\rangle ,\\ \langle [0.5,0.6],0.3\rangle \end{array}\right)$	$\left(\begin{array}{c} \langle [0.6,0.8],0.6\rangle ,\\ \langle [0.4,0.5],0.4\rangle \end{array}\right)$
$v_{4}$	$\left(\begin{array}{c} \langle [0.5,0.7],0.5\rangle ,\\ \langle [0.6,0.7],0.6\rangle \end{array}\right)$	$\left(\begin{array}{c} \langle [0.7,0.8],0.4\rangle ,\\ \langle [0.3,0.5],0.6\rangle \end{array}\right)$	$\left(\begin{array}{c} \langle [0.7,0.8],0.6\rangle ,\\ \langle [0.4,0.5],0.6\rangle \end{array}\right)$	$\left(\begin{array}{c} \langle [0.6,0.8],0.9\rangle ,\\ \langle [0.4,0.6],0.3\rangle \end{array}\right)$
***Step 3.*** Utilizing the Equation (1) (or Equation (2)) of the PCFFMM (or PCFFGMM) operator each normalized PCFN decision matrices $N^{\left(s\right)}={\left[\alpha_{ij}^{\left(s\right)}\right]}_{4\times 4}\;(s=1,2,3)$ are aggregated into the overall normalized PCFN decision matrix $N={\left[\alpha_{ij}\right]}_{4\times 4}$ for ϑ=2 and vector parameter r=(1,1,1) and given by Table 8

**Table 8:** The collective normalized PCFN decision matrix *R*.

	$C_{1}$	$C_{2}$	$C_{3}$	$C_{4}$
$v_{1}$	$\left(\begin{array}{c} \langle [0.7725,0.8148],0.8442\rangle ,\\ \langle [0.4662,0.5386],0.5073\rangle \end{array}\right)$	$\left(\begin{array}{c} \langle [0.7491,0.8116],0.7725\rangle ,\\ \langle [0.4713,0.5607],0.5607\rangle \end{array}\right)$	$\left(\begin{array}{c} \langle [0.7929,0.8567],0.7847\rangle ,\\ \langle [0.4142,0.4834],0.5050\rangle \end{array}\right)$	$\left(\begin{array}{c} \langle [0.7627,0.8180],0.8262\rangle ,\\ \langle [0.4834,0.5548],0.4978\rangle \end{array}\right)$
$v_{2}$	$\left(\begin{array}{c} \langle [0.7282,0.7847],0.7676\rangle ,\\ \langle [0.4713,0.5412],0.4662\rangle \end{array}\right)$	$\left(\begin{array}{c} \langle [0.6726,0.7369],0.7956\rangle ,\\ \langle [0.5561,0.6759],0.5246\rangle \end{array}\right)$	$\left(\begin{array}{c} \langle [0.7572,0.8364],0.7958\rangle ,\\ \langle [0.4361,0.4888],0.5145\rangle \end{array}\right)$	$\left(\begin{array}{c} \langle [0.79560.8364],0.8057\rangle ,\\ \langle [0.3632,0.4888],0.5222\rangle \end{array}\right)$
$v_{3}$	$\left(\begin{array}{c} \langle [0.6726,0.7533],0.7415\rangle ,\\ \langle [0.4866,0.5798],0.5222\rangle \end{array}\right)$	$\left(\begin{array}{c} \langle [0.7230,0.7847],0.7676\rangle ,\\ \langle [0.4422,0.5412],0.5050\rangle \end{array}\right)$	$\left(\begin{array}{c} \langle [0.7676,0.8499],0.7627\rangle ,\\ \langle [0.4713,0.5412],0.4715\rangle \end{array}\right)$	$\left(\begin{array}{c} \langle [0.7676,0.8262],0.8057\rangle ,\\ \langle [0.4361,0.5050],0.4834\rangle \end{array}\right)$
$v_{4}$	$\left(\begin{array}{c} \langle [0.7071,0.7956],0.7725\rangle ,\\ \langle [0.4888,0.6026],0.5430\rangle \end{array}\right)$	$\left(\begin{array}{c} \langle [0.8090,0.8499],0.7793\rangle ,\\ \langle [0.3977,0.4713],0.5050\rangle \end{array}\right)$	$\left(\begin{array}{c} \langle [0.7847,0.8499],0.8409\rangle ,\\ \langle [0.4185,0.5050],0.4441\rangle \end{array}\right)$	$\left(\begin{array}{c} \langle [0.7174,0.7958],0.8506\rangle ,\\ \langle [0.4993,0.5798],0.4843\rangle \end{array}\right)$
***Step 4.*** The overall evaluation values $\left\{\alpha_{1},\alpha_{2},\alpha_{3},\alpha_{4}\right\}$ relative to each choice $v_{i}\;(i=1,2,3,4)$ using PCFFMM operator with parameter ϑ=2 and vector parameter r=(1,1,1,1) by aggregating the preference values $\alpha_{ij}$ of $N={\left[\alpha_{ij}\right]}_{4\times 4}$ are given by:
  (4) $$\begin{array}{cc} \alpha_{1}=\left(\begin{array}{c} \langle [0.7965,0.8146],0.8090\rangle ,\\ \langle [0.5467,0.5706],0.5654\rangle \end{array}\right)\; & \alpha_{2}=\left(\begin{array}{c} \langle [0.7881,0.8069],0.8034\rangle ,\\ \langle [0.5448,0.5741],0.5620\rangle \end{array}\right)\\ \alpha_{3}=\left(\begin{array}{c} \langle [0.7861,0.8083],0.7968\rangle ,\\ \langle [0.5469,0.5730],0.5584\rangle \end{array}\right)\; & \alpha_{4}=\left(\begin{array}{c} \langle [0.7931,0.8142],0.81063\rangle ,\\ \langle [0.5439,0.5718],0.5577\rangle \end{array}\right) \end{array}\}$$
  Again, utilizing the PCFFGMM operator the aggregated values $\alpha_{i}\;(i=1,2,3,4)$ corresponding to each choice $v_{i}\;(i=1,2,3,4)$ for parameter ϑ=2 and vector parameter r=(1,1,1,1) are calculated as follows:
  (5) $$\begin{array}{cc} \alpha_{1}=\left(\begin{array}{c} \langle [0.5696,0.5840],0.5863\rangle ,\\ \langle [0.7744,0.7949],0.7901\rangle \end{array}\right)\; & \alpha_{2}=\left(\begin{array}{c} \langle [0.5440,0.5689],0.5626\rangle ,\\ \langle [0.7905,0.8125],0.8031\rangle \end{array}\right)\\ \alpha_{3}=\left(\begin{array}{c} \langle [0.5432,0.5704],0.5540\rangle ,\\ \langle [0.7909,0.8108],0.8023\rangle \end{array}\right)\; & \alpha_{4}=\left(\begin{array}{c} \langle [0.5494,0.5778],0.5833\rangle ,\\ \langle [0.7885,0.8097],0.8123\rangle \end{array}\right) \end{array}\}$$
***Step 5.*** The score values $S^{i}=S(\alpha_{i})\;(i=1,2,3,4)$ of evaluations $\alpha_{i}\;(i=1,2,3,4)$ in Equation (4) as obtained by the PCFFMM are calculated as follows:
  $$S^{1}=0.0376,\;S^{2}=0.0352,\;S^{3}=0.0357,\;S^{4}=0.0359.$$
  Since $S^{1}>S^{4}>S^{3}>S^{2}$, therefore the order of preference of the choices is $v_{1}≻v_{4}≻v_{3}≻v_{2}$. Thus the best choice is the alternative $v_{1}$. Again, the score values $S^{i}=S(\alpha_{i})\;(i=1,2,3,4)$ of the overall evaluations $\alpha_{i}\;(i=1,2,3,4)$ in Equation (5) as obtained by the PCFFGMM operator are calculated as follows:
  $$S^{1}=-0.0317,\;S^{2}=-0.0374,\;S^{3}=-0.0362,\;S^{4}=-0.0372.$$
  Since $S^{1}>S^{3}>S^{4}>S^{2}$, therefore the order of preference of the choices is $v_{1}≻v_{3}≻v_{4}≻v_{2}$. Thus the best choice is the choice $v_{1}$. Although, the ranking order of choices $v_{i}\;(i=1,2,3,4)$ determined by the PCFFMM and PCFFGMM operators differ due to the positions of choices $v_{3}$ and $v_{4}$. However, both the PCFFMM and PCFFGMM operator lead to the same best choice $v_{1}$.

### 6.1 Impact of the parameter *ϑ* during MCDM

In the following, an investigation is performed to examine the effect of *ϑ* on the ordering of choices using PCFFMM and PCFFGMM operators. For various values of *ϑ*, and vector parameter r=(1,1,1,1) ranking of the choices $v_{i}\;(i=1,2,3,4)$ are determined by PCFFMM as given by Table 9.

**Table 9:** Ranking of choices using PCFFMM operator based on the parameter *ϑ*.

Parameter *ϑ*	Score values	Ranking order of choices
2	$\begin{array}{c} S^{1}=0.0376,\;S^{2}=0.0352,\\ S^{3}=0.0357,\;S^{3}=0.0359 \end{array}$	$v_{1}≻v_{4}≻v_{3}≻v_{2}$
5	$\begin{array}{c} S^{1}=0.0423,\;S^{2}=0.0363,\\ S^{3}=0.0416,\;S^{4}=0.0421 \end{array}$	$v_{1}≻v_{4}≻v_{3}≻v_{2}$
10	$\begin{array}{c} S^{1}=0.0461,\;S^{2}=0.0421,\\ S^{3}=0.0455,\;S^{4}=0.0459 \end{array}$	$v_{1}≻v_{4}≻v_{3}≻v_{2}$
20	$\begin{array}{c} S^{1}=0.0493,\;S^{2}=0.0433,\\ S^{3}=0.0490,\;S^{4}=0.0492 \end{array}$	$v_{1}≻v_{4}≻v_{3}≻v_{2}$
50	$\begin{array}{c} S^{1}=0.0527,\;S^{2}=0.0467,\\ S^{3}=0.0523,\;S^{4}=0.0525 \end{array}$	$v_{1}≻v_{4}≻v_{3}≻v_{2}$

**Figure 1:**
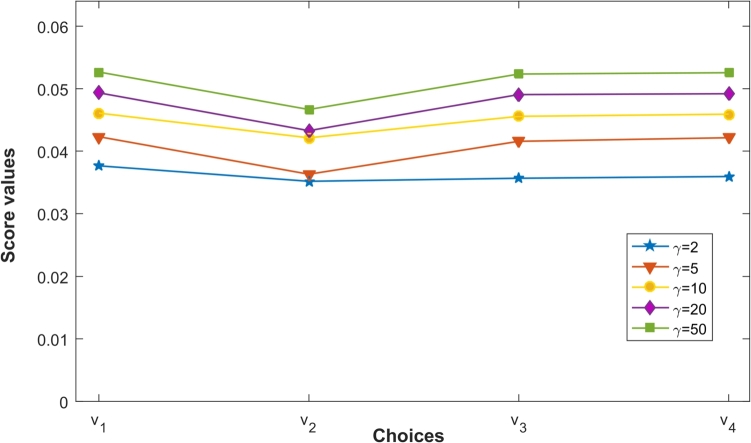
Ranking of choices obtained by the PCFFMM operator for different parameter *ϑ*.

It has been observed that in Table 9 and Fig. 1 score values of choices $v_{i}\;(i=1,2,3,4)$ determined by PCFFMM increases with the parameter *ϑ*. However, ranking order of the choices $v_{i}\;(i=1,2,3,4)$ remains unchanged as $v_{1}≻v_{4}≻v_{3}≻v_{2}$ despite of variation of parameter *ϑ*.

Again, the ranking order of choices $v_{i}\;(i=1,2,3,4)$ are obtained by PCFFGMM is given by Table 10.

**Table 10:** Ranking of choices using PCFFGMM based on the parameter *ϑ*.

*ϑ*	Score values	Ranking order of choices
2	$\begin{array}{c} S^{1}=-0.0387,\;S^{2}=-0.0415,\\ S^{3}=-0.0402,\;S^{4}=-0.0407 \end{array}$	$v_{1}≻v_{3}≻v_{4}≻v_{2}$
5	$\begin{array}{c} S^{1}=-0.0423,\;S^{2}=-0.0435,\\ S^{3}=-0.0428,\;S^{4}=-0.0430 \end{array}$	$v_{1}≻v_{3}≻v_{4}≻v_{2}$
10	$\begin{array}{c} S^{1}=-0.0461,\;S^{2}=-0.0474,\\ S^{3}=-0.0462,\;S^{4}=-0.0465 \end{array}$	$v_{1}≻v_{3}≻v_{4}≻v_{2}$
20	$\begin{array}{c} S^{1}=-0.0493,\;S^{2}=-0.0506,\\ S^{3}=-0.0494,\;S^{4}=-0.0496 \end{array}$	$v_{1}≻v_{3}≻v_{4}≻v_{2}$
50	$\begin{array}{c} S^{1}=-0.0526,\;S^{2}=-0.0538,\\ S^{3}=-0.0527,\;S^{4}=-0.0528 \end{array}$	$v_{1}≻v_{3}≻v_{4}≻v_{2}$

**Figure 2:**
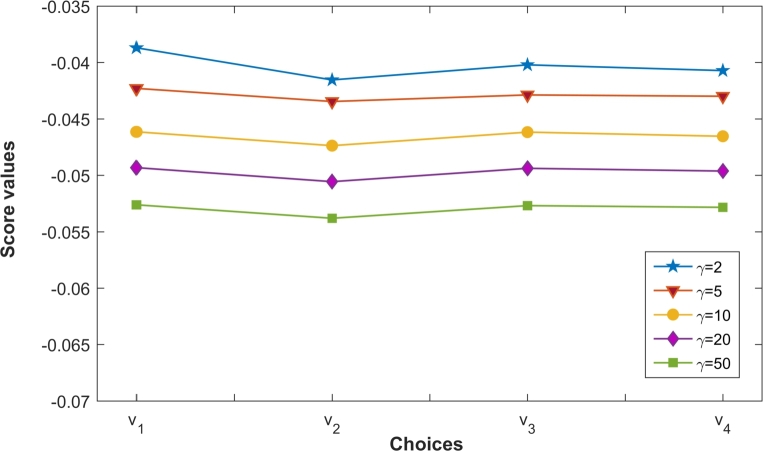
Ranking of choices obtained by the PCFFGMM operator for different parameter *ϑ*.

Again, Table 10 and Fig. 2 indicate that scores of choices $v_{i}$ utilizing PCFFGMM decreases with parameter *ϑ*. However, the ranking order of choices $v_{i}\;(i=1,2,3,4)$ remains unchanged as $v_{1}≻v_{3}≻v_{4}≻v_{2}$ for various values of *ϑ*. However, both PCFFMM and PCFFGMM result in same best alternative is $v_{1}$. This reflects the stability and robustness of the proposed operators PCFFGMM and PCFFGMM during MCDM.

### 6.2 Impact of the vector parameter r during MCDM

Here, the effect of r on overall ordering of choices using PCFFMM and PCFFGMM is presented. For various values of r, and ϑ=2 the ranking order of choices $v_{i}\;(i=1,2,3,4)$ are determined by PCFFMM is given by Table 11.

**Table 11:** Ranking of choices using PCFFMM for *ϑ* = 2 and different vector parameters *r*.

Vector parameter r	Score values	Ranking order of choices
r=(1,0,0,0)	$\begin{array}{c} S^{1}=-0.0737,\;S^{2}=-0.0763,\\ S^{3}=-0.0760,\;S^{4}=-0.0756 \end{array}$	$v_{1}≻v_{4}≻v_{3}≻v_{2}$
r=(1,1,0,0)	$\begin{array}{c} S^{1}=-0.0150,\;S^{2}=-0.0183,\\ S^{3}=-0.0177,\;S^{4}=-0.0174 \end{array}$	$v_{1}≻v_{4}≻v_{3}≻v_{2}$
r=(1,1,1,0)	$\begin{array}{c} S^{1}=0.0179,\;S^{2}=0.0150,\\ S^{3}=0.0156,\;S^{4}=0.0159 \end{array}$	$v_{1}≻v_{4}≻v_{3}≻v_{2}$
*r* = (1,1,1,1)	$\begin{array}{c} S^{1}=0.0376,\;S^{2}=0.0352,\\ S^{3}=0.0356,\;S^{4}=0.0359 \end{array}$	$v_{1}≻v_{4}≻v_{3}≻v_{2}$
r=(2,0,0,0)	$\begin{array}{c} S^{1}=0.0168,\;S^{2}=0.0126,\\ S^{3}=0.0134,\;S^{4}=0.0138 \end{array}$	$v_{1}≻v_{4}≻v_{3}≻v_{2}$

It is observed that in Table 11 overall ordering of choices $v_{i}\;(i=1,2,3,4)$ determined by PCFFMM for various values of r with ϑ=2, remains unchanged as $v_{1}≻v_{4}≻v_{3}≻v_{2}$.

Again, the ranking order of choices $v_{i}\;(i=1,2,3,4)$ obtained by PCFFGMM for various values of r with ϑ=2 is given by Table 12.

**Table 12:** Ranking of choices using PCFFGMM for *ϑ* = 2 and different vector parameters r.

Vector parameter r	Score values	Ranking order of choices
r=(1,0,0,0)	$\begin{array}{c} S^{1}=0.0754,\;S^{2}=0.0737,\\ S^{3}=0.0740,\;S^{4}=0.0738 \end{array}$	$v_{1}≻v_{3}≻v_{4}≻v_{2}$
r=(1,1,0,0)	$\begin{array}{c} S^{1}=0.0170,\;S^{2}=0.0151,\\ S^{3}=0.0156,\;S^{4}=0.0152 \end{array}$	$v_{1}≻v_{3}≻v_{4}≻v_{2}$
r=(1,1,1,0)	$\begin{array}{c} S^{1}=-0.0162,\;S^{2}=-0.0178,\\ S^{3}=-0.0174,\;S^{4}=-0.0177 \end{array}$	$v_{1}≻v_{3}≻v_{4}≻v_{2}$
r=(1,1,1,1)	$\begin{array}{c} S^{1}=-0.0317,\;S^{2}=-0.0374,\\ S^{3}=-0.0362,\;S^{4}=-0.0372 \end{array}$	$v_{1}≻v_{3}≻v_{4}≻v_{2}$
r=(2,0,0,0)	$\begin{array}{c} S^{1}=-0.0143,\;S^{2}=-0.0165,\\ S^{3}=-0.0159,\;S^{4}=-0.0164 \end{array}$	$v_{1}≻v_{3}≻v_{4}≻v_{2}$

**Figure 3:**
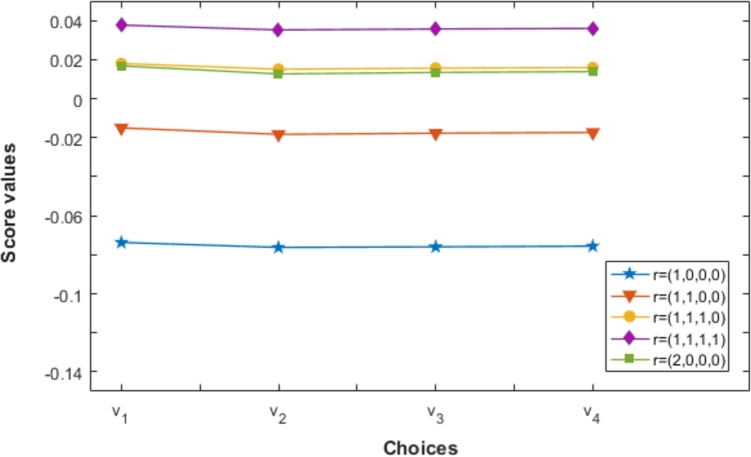
Ranking of choices by PCFFMM for different vector parameters r at *ϑ* = 2.

**Figure 4:**
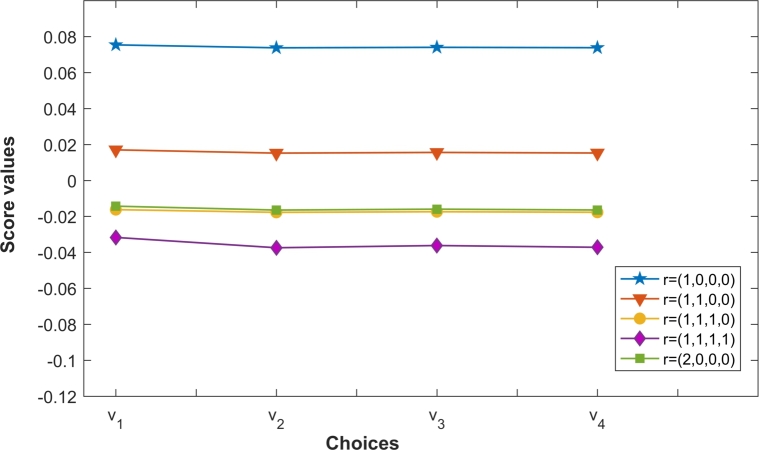
Ranking of choices by PCFFGMM for different vector parameters r at *ϑ* = 2.

Again, from Table 12 the overall ranking of choices $v_{i}\;(i=1,2,3,4)$ determined by the PCFFGMM for various values of r with ϑ=2 is always $v_{1}≻v_{3}≻v_{4}≻v_{2}$. Similarly for various values of *r* the overall ranking of choices $v_{i}\;(i=1,2,3,4)$ at ϑ=5,10,20,50 remains the same as $v_{1}≻v_{4}≻v_{3}≻v_{2}$. This reflects the stability of the proposed operators PCFFMM and PCFFGMM during MCDM. Also, Figure 3, Figure 4 are depicting the variation of ranking order of choices for different vector parameters r and establishing the fact that the variation of the vector parameters r doesn't alter the best choice $v_{1}$.

### 6.3 A relative analysis of the projected approach with some existent MCDM approach

Here, a relative investigation of the projected MCDM approach with some existent MCDM approach is presented. The comparison results are obtained by applying the existing methods [74], [78] to the above example, which is given by Table 13.

**Table 13:** A comparative study with several MCDM approaches.

MCDM Method	Aggregation Operator	Ranking order of choices
Khan et al. [74]	PCFWA [74]	$v_{1}≻v_{3}≻v_{2}≻v_{4}$
Khan et al. [74]	PCFWG [74]	$v_{1}≻v_{2}≻v_{3}≻v_{4}$
Hussain et al. [78]	PFEWG [78]	$v_{1}≻v_{2}≻v_{4}≻v_{3}$
Proposed method	PCFFMM	$v_{1}≻v_{4}≻v_{3}≻v_{2}$
Proposed method	PCFFGMM	$v_{1}≻v_{3}≻v_{4}≻v_{2}$

The ranking results due to Khan et al. [74] and Hussain et al. [78] have the same best choice $v_{1}$ as the proposed method. However, the existing methods by Khan et al. [74] and Hussain et al. [78] don't take into consideration interrelationship among the criteria whereas handling of the interrelationship among the criteria is a prominent feature of the projected method. The operators PCFFMM and PCFFGMM are based on Frank triangular norms, which are much adaptable and robust as compare to other families of triangular norms, also they can reflect the interrelationship among the criteria. Further, PCFMM and PCFFGMM are some extensions of Pythagorean Muirhead mean operators which not only deal with the interactions among the criteria but also can efficaciously address the aspects as demonstrated by Xu et al. [28]. Furthermore, the parameter r serves a crucial part while solving MCDM problems. The influence of the parameter vector r is explained in Section 6.2. From Table 3, Table 4, it has been observed that by allotting different values to *r* the overall ranking result of the choices $v_{i}\;(i=1,2,3,4)$ determined by PCFFMM or PCFFGMM operator varies based on their score values, however, the best choice remains the same. The difference in ranking of Khan et al. [74], Hussain et al. [78] and the projected approach is due to consideration of interrelationship among the criteria and influence of parameters ϑ,r in the proposed decision-making method which not considered by Khan et al. [74], and Hussain et al. [78]. Hence, it is expected that the projected approach using PCFFMM and PCFFGMM might be more effective than the existing methods [74], [78].

### 6.4 Benefits of the projected MCDM approach

Some of the benefits of the projected MCDM approach are as follows:

(1) The projected MCDM approach is based on Pythagorean cubic fuzzy sets (PCFSs) [74], which can depict the ambiguities information often involved in practical problems. The PCFS [74] can express the imprecise and vague information more appropriately as they integrate the IVPFS and PFS concurrently. Thus the projected MCDM approach based on PCFSs can furnish much adaptability and robustness towards complex decision-making problems.
(2) The PCFFMM and PCFFGMM proposed in this paper can provide a flexible and robust overall result due to the existence of a parameter vector. Further, the PCFFMM and PCFFGMM operators are based on Frank triangular norms [79] which is a family of the continuous triangular norm and provides more flexibility than Algebraic and the Einstein triangular norms due to the presence of an additional parameter.
(3) The proposed PCFFMM and PCFFGMM operators can handle the dependency between several supplied arguments and also provide more flexibility and robustness during MCDM. The BM [23] and MSM [35] which considers interaction among the criteria are the special cases of the MM [25]. From Corollary 4.3, Corollary 4.4 it has been observed that the PCFFMM and PCFFGMM can be reduced to the PCFFMSM and PCFFGMSM. Also, from Corollary 4.5, Corollary 4.6 it is shown that the PCFFMM and PCFFGMM operators can be restricted to the PCFFBM and PCFFGBM. Moreover, PCFFMM and PCFFGMM have less computation complexity as compared to the Choquet integral [92] based aggregation operators. Hence the proposed PCFFBM and PCFFGBM can provide more general and robust results during complex decision-making situations.
(4) The proposed MCDM method incorporated with the PCFFMM and PCFFGMM can provide a more precise result for complex real-life problems with multiple interrelated criteria under the PCFS environment. Flexibility and robustness of collective outcomes of an MCDM problem are due to the strength of PCFFMM and PCFFGMM operators during the aggregation process. The benefits of the projected MCDM approach over some of the existing approach [74], [78] have been described in Section 6.3.

## 7 Conclusion

In this study, two new Pythagorean cubic fuzzy Frank Muirhead mean operators viz., the PCFFMM, and PCFFGMM are proposed. Further, Some of their particular instances and features are studied. An approach MCDM has been developed, which allows interaction among the criteria with the Pythagorean cubic fuzzy information. A demonstration of the projected MCDM approach is presented and a relative study with a few existent approaches is provided.

Some major findings are given as:

(1) The present study introduces some novel Muirhead mean [25] operators such as the PCFFMM and PCFFGMM under Frank *t*-norms based on PCFSs [74] to interpret the vagueness and imprecision of practical problems.
(2) One of the salient features of PCFFMM and PCFFGMM operators is the ability to reflect the interrelationships among multiple arguments. These proposed operators can afford much adaptability and robustness during the MCDM process due to the occurrence of a parameter vector. Also, the proposed PCFFMM and PCFFGMM operators represent a flexible class of aggregation operators because Frank triangular norms [79] can provide more flexibility than some of the existing operators [74], [75], [76], [77], [78] based on Algebraic and the Einstein triangular norms due to the presence of an additional parameter.
(3) The proposed MCDM method along with the PCFFMM and PCFFGMM operators can provide a more accurate result as compared to some of the existing MCDM methods [74], [78] for real-life problems with correlated criteria under the Pythagorean cubic fuzzy environment. The relevancy of the projected MCDM approach is manifested by an exemplifying example. Further, a relative analysis of the projected MCDM approach with some of the existing methods [74], [78] is presented to establish the effectiveness of the projected MCDM approach.

The projected MCDM approach may be further enhanced to analyze decision-making process under cubic linguistic Pythagorean fuzzy information. Moreover, the projected approach seems to be applicable to several practical fields like *Population survey*, *Medical diagnosis*, *Big data analytic*, *Industrial engineering*, and *Risk assessment*, etc. For future research direction the proposed MCDM method may be extended to study various emerging regions, e.g. *Optimization*, *Neural networks*, and *Image processing*, etc.

## Declarations

### Author contribution statement

**Pankaj Kakati:** Conceived and designed the experiments; Performed the experiments; Analyzed and interpreted the data; Contributed reagents, materials, analysis tools or data; Wrote the paper.

### Funding statement

This research did not receive any specific grant from funding agencies in the public, commercial, or not-for-profit sectors.

### Data availability statement

No data was used for the research described in the article.

### Declaration of interests statement

The authors declare no conflict of interest.

### Additional information

No additional information is available for this paper.
